# Neural interfaces: Bridging the brain to the world beyond healthcare

**DOI:** 10.1002/EXP.20230146

**Published:** 2024-03-14

**Authors:** Shumao Xu, Yang Liu, Hyunjin Lee, Weidong Li

**Affiliations:** ^1^ Department of Biomedical Engineering The Pennsylvania State University Pennsylvania USA; ^2^ Brain Health and Brain Technology Center at Global Institute of Future Technology Shanghai Jiao Tong University Shanghai China

**Keywords:** decoded neural activity, human‐machine interactions, mind communication, neural interfaces, remote control, smart homes

## Abstract

Neural interfaces, emerging at the intersection of neurotechnology and urban planning, promise to transform how we interact with our surroundings and communicate. By recording and decoding neural signals, these interfaces facilitate direct connections between the brain and external devices, enabling seamless information exchange and shared experiences. Nevertheless, their development is challenged by complexities in materials science, electrochemistry, and algorithmic design. Electrophysiological crosstalk and the mismatch between electrode rigidity and tissue flexibility further complicate signal fidelity and biocompatibility. Recent closed‐loop brain‐computer interfaces, while promising for mood regulation and cognitive enhancement, are limited by decoding accuracy and the adaptability of user interfaces. This perspective outlines these challenges and discusses the progress in neural interfaces, contrasting non‐invasive and invasive approaches, and explores the dynamics between stimulation and direct interfacing. Emphasis is placed on applications beyond healthcare, highlighting the need for implantable interfaces with high‐resolution recording and stimulation capabilities.

## Graphical abstract

This perspective explores diverse neural interfaces in the brain, offering transformative possibilities for engaging with our environment and enhancing communication. Emphasis is placed on the role of both non‐invasive and invasive neural interfaces in transforming practical applications beyond the realm of healthcare, emphasizing the promising implications for remote control, mind connectivity, and integrated urban design.

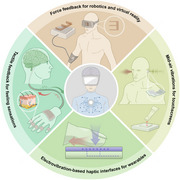

## 1 INTRODUCTION

With over 86 billion neurons and trillions of connections, the human brain is a complex organ, characterized by its remarkable capacity for processing, learning, and adapting.^[^ ^1^, ^2^, ^3^, ^4^, ^5^, ^6^, ^7^ ^]^ This has led to significant interest in brain‐machine interfaces (BMIs) and brain‐computer interfaces (BCIs),^[^ ^8^, ^9^, ^10^, ^11^ ^]^ which are transforming how we interact with our surroundings and communicate.^[^ ^12^ ^]^ By interpreting brain activity, these technologies enable intuitive and natural manipulation of external devices.^[^ ^13^, ^14^ ^]^ High‐resolution and reliable neural interfaces are paving the way for direct brain‐to‐device and brain‐to‐computer connection, heralding a new era of information exchange and thought communication.^[^ ^11^, ^15^ ^]^

Historically, neural interfaces have played a pivotal role in healthcare. Serving as an intermediary between external electronic devices and biological tissue, these interfaces, particularly neural microelectrodes, have been crucial in both recording bioelectrical signals for sensory information and motility mapping and in electrically stimulating neural tissues for biological function regulation, such as altering ion concentrations inside and outside the cell membrane and improving neural signal transmission.^[^ ^16^, ^17^, ^18^, ^19^, ^20^ ^]^ This advancement has deepened our insight into the workings of the nervous system and improved the treatment of neurological conditions like Parkinson's disease and epilepsy, as well as sensory impairments like hearing and vision loss.^[^ ^21^ ^]^

Early multi‐channel silicon‐based neural electrodes, like the Utah array and the Michigan probe, caused cell tissue death and inflammatory reactions, hampering signal stability.^[^ ^22^, ^23^ ^]^ The evolution towards flexible polymer microelectrode arrays has been a significant step forward, yet challenges remain in electrocorticography (ECoG) signal clarity due to tissue‐electrode interface mismatches.^[^ ^24^, ^25^ ^]^ Neural interfaces have undergone significant evolution, transcending their traditional medical applications to pioneer groundbreaking uses across various sectors. Central to these advancements is the ability to decode brain patterns, enabling control over external devices like prosthetics.^[^ ^10^, ^26^ ^]^ These interfaces offer an intuitive control, akin to the natural use of limbs, representing a breakthrough in assistive technology. The development of these interfaces involves direct measurement of brain activity with diverse temporal and spatial resolutions, combined with advanced mathematical modeling. Nevertheless, these technological advances face significant challenges. Precise control requires sophisticated algorithms capable of decoding complex neural signals.^[^ ^9^ ^]^ Additionally, ensuring long‐term biocompatibility and minimizing adverse biological responses, such as inflammation, fibrosis, infection, and neurodegeneration, is crucial for their safe and effective deployment.^[^ ^10^, ^27^ ^]^ Recent advancements in fully internalized microelectrodes for deep brain stimulation (DBS), cochlear implants, and retinal prostheses have been driven by designs that enhance safety and enable long‐term stimulation and recording.^[^ ^17^, ^28^, ^29^ ^]^

Beyond healthcare, these neural interfaces have the potential to transform fields such as virtual reality, smart home technology, and urban planning.^[^ ^30^, ^31^, ^32^, ^33^, ^34^, ^35^, ^36^, ^37^ ^]^ These interfaces offer immersive interactions with virtual worlds, improved Internet of Things (IoT) control,^[^ ^38^ ^]^ and the potential for emotion sharing and mind connectivity.^[^ ^39^ ^]^ Envision a future where self‐driving cars,^[^ ^40^ ^]^ smart homes,^[^ ^41^ ^]^ and other urban utilities are not just automated, but also directly controlled by brain signals,^[^ ^42^, ^43^ ^]^ resulting in an efficient and interconnected landscape.^[^ ^44^ ^]^

This perspective delves into the progress and challenges of neural interfaces, which are changing how we interact with our environment. These interfaces work by recording and decoding neural signals, thus enabling direct connections between the brain and devices for seamless information sharing and collective experiences. However, significant challenges also remain. These include the complexity of the algorithms required, the interference of electrophysiological signals, and the incompatibility between the hardness of the electrodes and the softness of neural tissue. This perspective also highlights the differences between non‐invasive and invasive neural interfaces. While non‐invasive methods are less risky and easier to use, invasive interfaces offer higher resolution and are more effective for specific applications. Advancements in high‐density biocompatible implantable interfaces are increasingly essential beyond healthcare applications like human‐machine interactions. The potential of neural interfaces in these areas is vast, but realizing this potential requires addressing the technical limitations and ethical concerns.

## 2 BRAIN NEURAL INTERFACE

Brain neural interfaces, such as electroencephalography (EEG), ECoG, subcortical microelectrode arrays (MEA), and DBS, offer unprecedented opportunities for healthcare and human‐machine interactions.

### 2.1 Brain regions and neural interfaces

**FIGURE 1:**
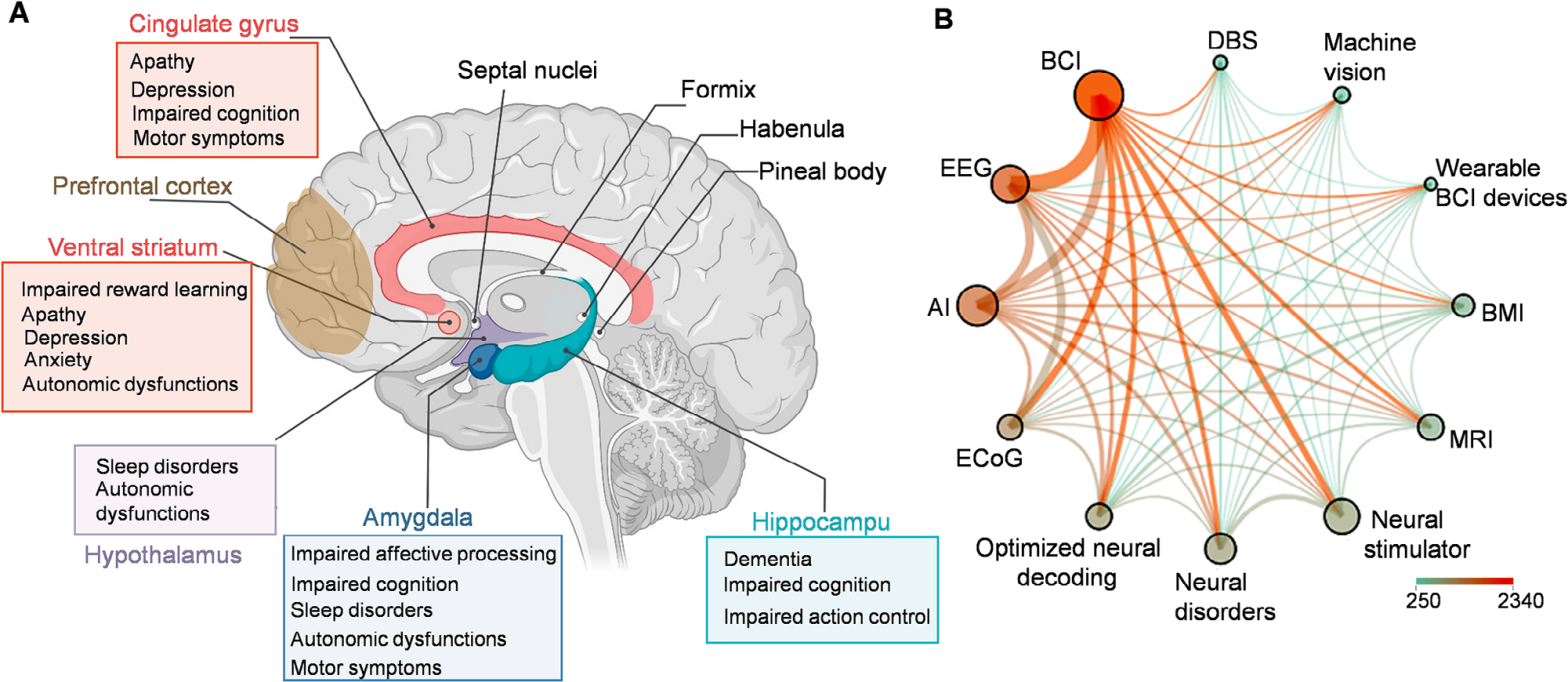
(A) Representation of selected brain regions and their associated clinical symptoms. A, created by Biorender.com. (B) Top keywords in the neural interface publications.

**FIGURE 2:**
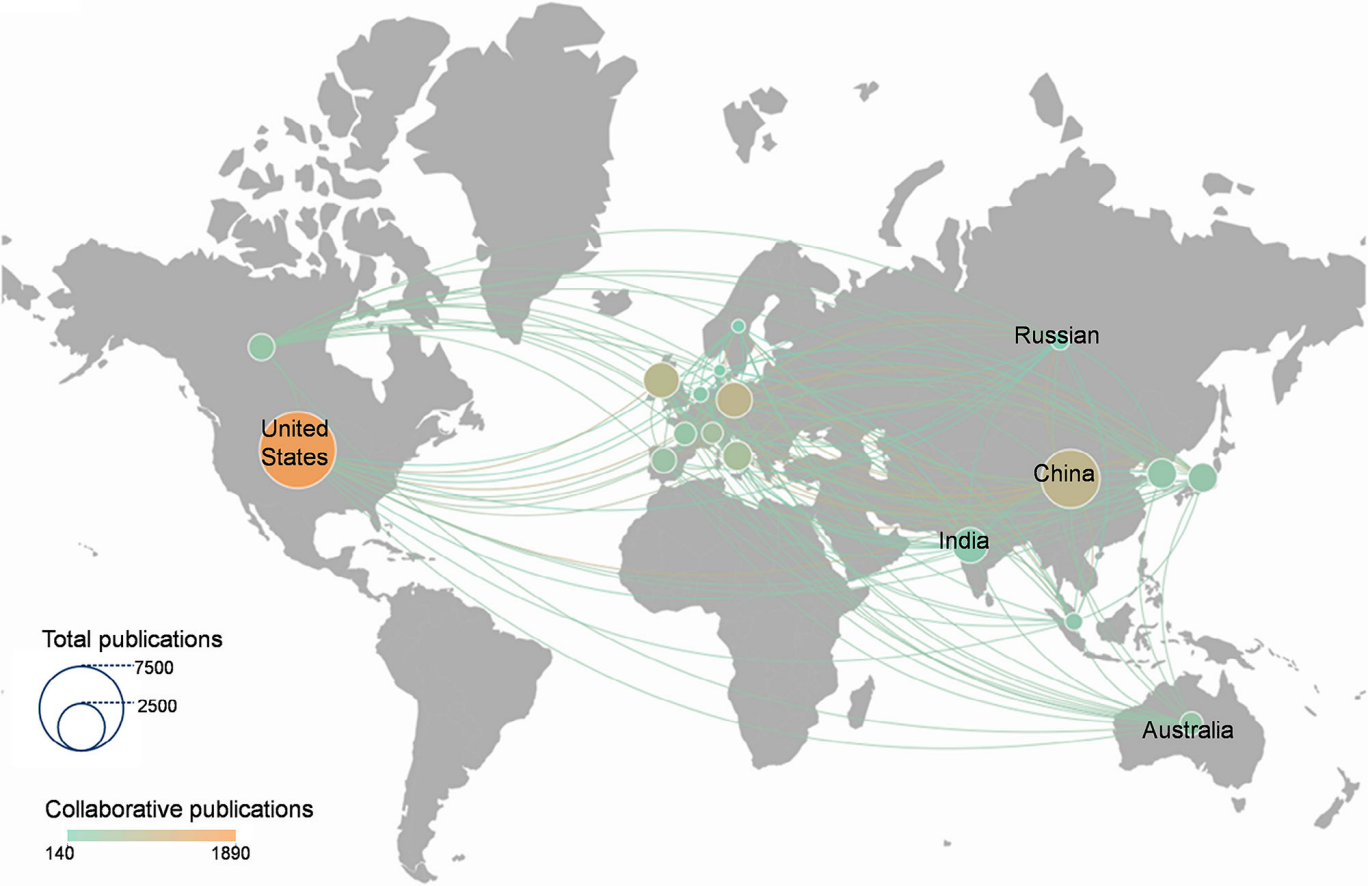
Publications with the subject of “neural interface” in different countries. A bibliometric approach and network analysis were employed to visualize the collaboration patterns. The size of the circles on the map corresponds to the total number of publications from each country, while different colors indicate collaborative publications.

The brain is an intricate organ with various regions in charge of thoughts, emotions, and behaviors.^[^ ^45^, ^46^, ^47^, ^48^, ^49^ ^]^ The prefrontal cortex, often considered the “thinking brain”, is primarily accountable for executive functions like decision‐making and planning^[^ ^50^ ^]^ (Figure 1A). Regions like the cingulate gyrus and ventral striatum are commonly referred to as the “emotional brain”, as they are critical in shaping our emotional experiences and responses.^[^ ^51^ ^]^ The amygdala, hypothalamus, and hippocampus are often described as the “doing brain”, with functions that include sleep regulation, autonomic control, and motor activities.^[^ ^52^, ^53^ ^]^ Techniques like DBS are powerful tools that target these specific regions, presenting potential treatments for mood disorders and other neural conditions. The field of neural interfaces is witnessing significant growth, especially in the areas of artificial intelligence (AI)‐driven neural decoding and neural stimulation therapies (Figure 1B). This expansion is marked by a diverse set of international collaborations, as indicated by various color‐coded clusters in research output (Figure 2). The United States leads in scholarly output on “neural interface”, followed by China, Germany, and the United Kingdom. Among the top 20 contributing nations, approximately 30% of their published work involves international cooperation. This trend underscores a global, interdisciplinary effort in advancing neural interface science. The burgeoning neural interfaces hold promise not only for medical applications but also for broad societal impacts, especially as these technologies increasingly integrate into daily life.

### 2.2 Historical evolution in different neural interfaces

**FIGURE 3:**
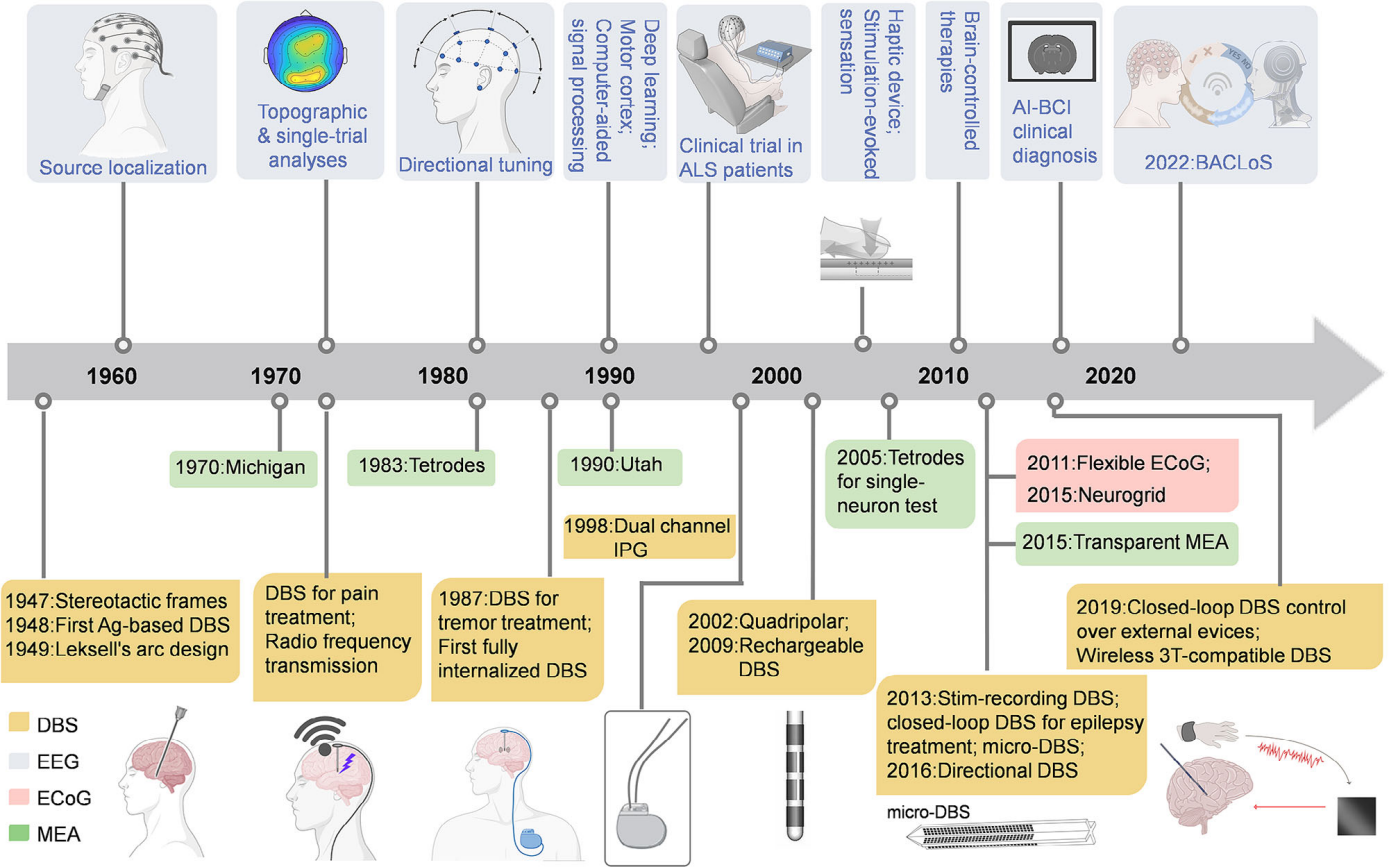
Evolution of different neural interface techniques. ALS, amyotrophic lateral sclerosis; Ag, silver; stim, stimulation; IPG, implantable pulse generator; BACLoS, brain‐AI closed‐loop system. Micro‐DBS, reproduced under the terms of the CC‐BY Creative Commons Attribution International license (https://creativecommons.org/licenses).^[^ ^87^ ^]^ Copyright 2019, The Authors, published by Frontiersin.org. Partially created by Biorender.com.

Starting in the 1960s, EEG advancements enabled brain source localization, such as identifying epilepsy foci,^[^ ^54^ ^]^ marking a significant leap in understanding and treating neurological conditions (Figure 3). The 1970s saw further progress with the development of methods for topographic analyses of EEG data, enhancing visualization of the spatial distribution of brain activity.^[^ ^55^, ^56^, ^57^ ^]^ Additionally, improvements in computational methods enabled single‐trial EEG data analyses, enhancing temporal resolution and precision.^[^ ^58^ ^]^ The 1980s were marked by directional tuning research, focusing on how a neuron firing rate changes with the movement direction,^[^ ^59^, ^60^ ^]^ which significantly enhanced our understanding of motor control and brain‐coordinated movement. The 1990s introduced deep learning algorithms in EEG data analysis^[^ ^61^, ^62^ ^]^ and saw the first clinical demonstrations of BCIs in humans, especially for individuals with amyotrophic lateral sclerosis (ALS), showcasing the potential of BCIs in augmenting communication and control.^[^ ^63^ ^]^ The 2000s brought about the development in sensory haptic devices^[^ ^64^ ^]^ and research into how stimulation affects sensation and perception,^[^ ^65^, ^66^ ^]^ providing insights into sensory processing. In the 2010s, the integration of brain‐controlled therapies and AI with BCIs for clinical diagnosis opened new pathways in treating neurological disorders.^[^ ^67^ ^]^ The emergence of the metaverse in 2021, integrating BCIs with virtual and augmented reality, followed by the development of the brain‐AI closed‐loop system (BACLoS) in 2022,^[^ ^68^ ^]^ has marked recent progress. ECoG has evolved significantly, with the development of foldable and flexible ECoG in 2011,^[^ ^69^ ^]^ and high‐density Neurogrid in 2015.^[^ ^70^ ^]^ The evolution of MEAs traces back to the early development of the Michigan silicon electrode which laid the groundwork for precise neural recording and stimulation. By 1983, tetrodes allowed for simultaneous recording from multiple neurons,^[^ ^71^ ^]^ and the 1990s saw the development of the Utah array, notable for its detailed brain mapping capabilities.^[^ ^72^ ^]^ In 2005, advancements in tetrode technology improved single‐neuron representations, allowing for precise studies of individual neural activities.^[^ ^73^ ^]^ The development of a transparent intracortical microprobe array in 2015 enabled simultaneous electrical recording and optical stimulation, further advancing neuroscience research.^[^ ^74^ ^]^

DBS has evolved from stereotactic frames in 1947 for precise brain targeting^[^ ^75^ ^]^ to the first silver‐based DBS in 1948^[^ ^76^ ^]^ and Leksell's arc‐based design in 1949^[^ ^77^ ^]^ (Figure 3). The 1970s introduced DBS for pain treatment^[^ ^78^ ^]^ and external DBS systems with handheld radio frequency transmitters.^[^ ^79^ ^]^ The 1980s saw the first fully internalized DBS systems,^[^ ^80^ ^]^ and by the late 1980s, DBS was successfully used for tremor treatment.^[^ ^81^ ^]^ The late 1990s and early 2000s saw the development of dual‐channel implantable pulse generators (IPGs)^[^ ^82^ ^]^ and commercialized Quadripolar electrodes, allowing for precise controlled stimulation.^[^ ^83^ ^]^ In 2009, rechargeable batteries were introduced,^[^ ^84^ ^]^ followed by advancements in 2013 with stimulation‐recording adaptive DBS in Parkinson's disease treatment,^[^ ^85^ ^]^ closed‐loop systems for epilepsy,^[^ ^86^ ^]^ and micro‐DBS probes.^[^ ^87^, ^88^ ^]^ The introduction of directional Quadripolar DBS electrodes in 2016, with their ability to shape the electric field through directional electrodes, offered targeted stimulation to enhance therapeutic efficacy.^[^ ^89^ ^]^ The year 2019 saw developments, with the introduction of closed‐loop DBS enabling control over external devices^[^ ^90^ ^]^ and the advent of wireless 3 Tesla‐compatible DBS systems with imaging techniques to enhance targeted precision and treatment efficacy.^[^ ^91^ ^]^

### 2.3 Neural interfaces: Current advances and challenges

DBS, primarily recognized for its role in treating neurological conditions, is now being explored in applications beyond healthcare, particularly to mitigate movement disorder symptoms like tremors and rigidity.^[^ ^92^, ^93^ ^]^ However, its invasive nature poses risks and challenges. ECoG, another invasive method, involves direct electrode placement on the brain surface to detect electrical signals. It shows promise in controlling prosthetic limbs and monitoring epileptic seizures.^[^ ^11^, ^94^ ^]^ Current endeavors aim to develop more flexible and chronic electrodes to broaden their applicability.^[^ ^95^, ^96^ ^]^ MEA has the capability to precisely monitor neural activity, capturing signals from neuron clusters. As with other invasive methods, concerns about their long‐term integration and potential health impacts persist. EEG stands apart as a non‐invasive technique for monitoring neural activity by placing electrodes on the scalp.^[^ ^97^ ^]^ It has been effectively used in various applications including navigating virtual environments and monitoring neural responses in situations like fatigued driving. Nevertheless, its relatively low signal‐to‐noise ratio can be a limitation. Advanced signal processing techniques like time‐frequency analysis and independent component analysis (ICA) are utilized to improve EEG data accuracy.^[^ ^98^, ^99^ ^]^ Despite the potential of various neural interfaces, challenges in ensuring their accuracy, reliability, and long‐term safety, particularly when integrated into daily urban life, remain critical concerns.

#### 2.3.1 Biocompatibility, durability, and efficiency

When foreign electrodes are implanted in the brain, they typically trigger a foreign body response, including inflammation and scarring, which can reduce the effectiveness of the interface over time.^[^ ^24^, ^100^, ^101^ ^]^ To enhance biocompatibility, recent advancements have focused on utilizing nanomaterials and coatings to reduce inflammation and scarring.^[^ ^24^, ^101^ ^]^ As electrodes are miniaturized to enhance spatial resolution and reduce tissue damage, they face challenges such as a decreased signal‐to‐noise ratio and increased impedance, which hinders efficient signal transmission.^[^ ^102^ ^]^ To improve the durability and mechanical adhesion of electrodes to tissue, various treatments, including microwave treatment^[^ ^103^ ^]^ and tough interfacial covalent bonding,^[^ ^104^, ^105^, ^106^ ^]^ have been employed. Furthermore, the development of 3D nanostructures and the use of durable materials like nanostructured platinum (Pt)^[^ ^107^ ^]^ and iridium oxide (IrO_x_)^[^ ^108^ ^]^ have proven effective in improving long‐term stability. Over time, electrodes also suffer from degradation due to mechanical strain and continual cyclic loads, leading to cracking, delamination, and potential failure.^[^ ^17^ ^]^ To address these challenges, efforts have been made to optimize efficiency by balancing miniaturization with efficient signal transmission. This includes advancements in material engineering and electrode design, aiming to resolve issues related to decreased signal‐to‐noise ratio and increased impedance.^[^ ^109^ ^]^

#### 2.3.2 Crosstalk in high‐density electrode arrays

Crosstalk in high‐density electrode arrays presents a significant challenge in neural recordings and stimulation.^[^ ^24^, ^110^, ^111^, ^112^ ^]^ This issue arises from the proximity of electrodes within the array, causing their electric fields to overlap in time and space at the electrode and tissue interface. Crosstalk can significantly interfere with signal clarity and may lead to complex and adverse neural interactions. Crosstalk exceeding 1% is not negligible in neural signal recording,^[^ ^24^, ^110^ ^]^ as it can induce neural interactions and even inhibit neural activation if the extracellular potential exceeds the inhibition threshold. This limits the spatial‐temporal resolution and can adversely affect nearby sites in high‐density electrode arrays.^[^ ^24^ ^]^ Additionally, crosstalk issues may be exacerbated in flexible polymer arrays due to insulation limitations of polymer substrate and encapsulation layers.^[^ ^111^ ^]^

To overcome this, the design of electrode arrays is being refined by adjusting both electrode spacing and diameter.^[^ ^113^ ^]^ Increasing the distance between electrodes could reduce the overlap of their electric fields, thereby minimizing crosstalk.^[^ ^113^ ^]^ This spacing is fine‐tuned based on application needs and the specific neural tissue targeted, balancing the need for high spatial resolution with reduced interference. Additionally, reducing the diameter of electrodes limits the spatial extent of electric fields, further reducing crosstalk potential.^[^ ^113^ ^]^ However, smaller electrodes could result in increased impedance, thereby necessitating a balance to minimize crosstalk while simultaneously maintaining signal quality. Furthermore, the development of multi‐channel sites on electrode arrays represents a significant step forward.^[^ ^114^, ^115^ ^]^ These arrays could uniformly distribute electric fields across the surface, thereby reducing edge effects where electric field density tends to concentrate at the edge of the electrode.^[^ ^18^, ^114^ ^]^ This uniformity to address the crosstalk in high‐density electrode arrays leads to a more consistent modified coating, improving both the accuracy of electrode recordings and the efficacy of stimulation.

#### 2.3.3 Stability

The enduring functionality and effectiveness of neural interfaces are closely associated with the interface stability between electrodes and neural tissues. Challenges such as corrosion, dissolution, and material swelling at these electrode interfaces significantly impact the durability and operational performance of implants.^[^ ^22^, ^116^ ^]^ It is crucial to maintain interface stability and high‐quality electrochemical properties for long‐lasting and effective signal recording and neural stimulation. In response, material engineering has been a focal point. Conductive polymers like polypyrrole (PPy), polyaniline (PANI), poly(3,4‐ethylenedioxythiophene) (PEDOT), and poly(3‐hexylthiophene) (P3HT), renowned for their enhanced electrochemical properties and structural stability are utilized to prolong the life and reliability of neural interfaces by mitigating interface degradation.^[^ ^19^, ^117^ ^]^ Polydopamine (PDA) could enhance neural interfaces with its biocompatibility and adhesion, which promotes electrode–tissue integration, functionalization for neuron growth, reducing inflammation, and can be synergized with conductive polymers for a robust and biocompatible interface.^[^ ^19^, ^117^ ^]^ Moreover, the use of biomolecules, such as zwitterionic polymers for antifouling coatings,^[^ ^118^ ^]^ has significantly improved interface stability by effectively minimizing interface degradation. Additionally, electrochemical copolymerization could be also employed to create coatings that enhance electrode performance and durability.^[^ ^119^ ^]^ Furthermore, the development of biodegradable and flexible electrode materials like polylactic acid (PLA), polyglycolic acid (PGA), and polycaprolactone (PCL) offers adaptability to tissue deformation.^[^ ^120^ ^]^ Their biodegradability also contributes to minimizing adverse reactions, further enhancing interface functionality.

### 2.4 Monitoring EEG signals in real‐world applications beyond healthcare

**FIGURE 4:**
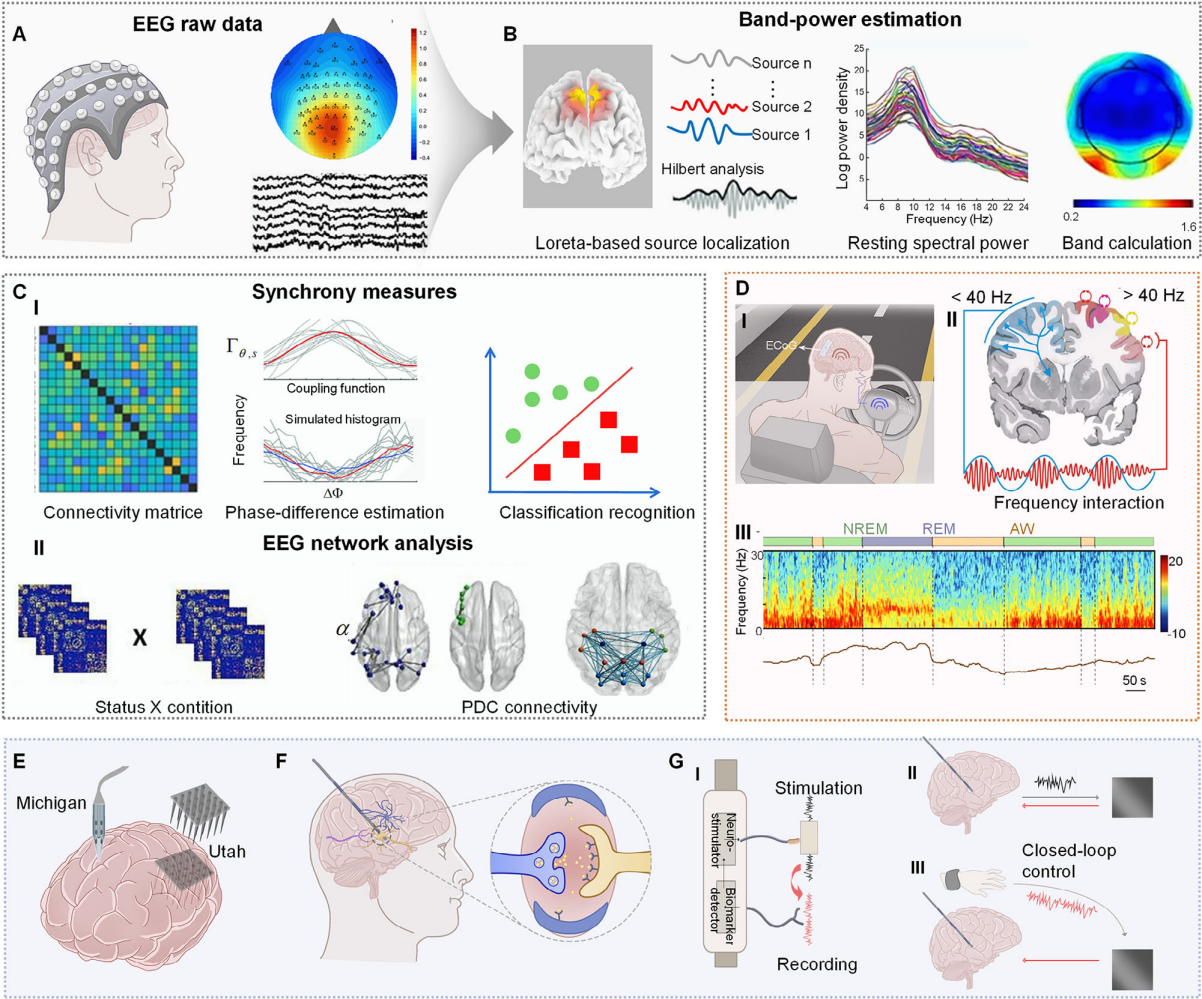
Neural interfaces monitoring and modulation. (A–C) EEG processing for neural signal monitoring. (A) Raw data collection. Reproduced with permission.^[^ ^121^ ^]^ Copyright 2013, Society for Neuroscience. (B) Band‐power estimation including Loreta localization, spectral power, and further calculation for the theta/alpha/beta bands. Reproduced with permission.^[^ ^121^, ^123^ ^]^ Copyright 2013, Society for Neuroscience; and permission.^[^ ^48^ ^]^ Copyright 2023, Elsevier. (C) Connectivity analyses. (I) Synchrony measures including connectivity matrices, phase‐difference estimation, and classification recognition. (II) EEG network analysis including group network connectivity and partial directed coherence (PDC) interactions. Reproduced with permission.^[^ ^123^, ^124^, ^134^ ^]^ Copyright 2022, Springer; 2023, Elsevier; and 2018, PLoS. (D) ECoG for neural signal monitoring. Reproduced under the terms of the CC‐BY Creative Commons Attribution 4.0 International license (https://creativecommons.org/licenses/by/4.0).^[^ ^145^, ^148^ ^]^ Copyright 2023, The Authors, published by Springer Nature; and reproduced with permission.^[^ ^69^ ^]^ Copyright 2011, IEEE. (I) ECoG grid and self‐driving application; (II) Gamma activity modulation of ECoG rhythms in local cortical processing. (III) Sleep fMRI: ECoG and BOLD signals. SWR: sharp wave ripples; NREM: non‐rapid eye movement; AW: awake. (E) MEA electrode. (F) DBS electrophysiological mechanism (calcium waves formation and gliotransmitters release with arteriole dilation and increased blood flow). (G) Closed‐loop DBS control. Reproduced with permission.^[^ ^90^, ^170^ ^]^ Copyright 2021 and 2019, Springer Nature. (I) Closed‐loop close model. (II) Sensing and stimulation through the same DBS electrodes. (III) Utilizing exterior sensing devices and stimulating.

EEG, which captures brain electrical activity using scalp‐placed electrodes, is becoming central to brain‐to‐device interactions, providing insights into the brain electrical activity and connectivity. EEG analyses are multi‐step processes including recording and preprocessing brain signals^[^ ^121^, ^122^ ^]^ (Figure 4A), identifying the power and specific frequency bands of these signals by band‐power estimation^[^ ^121^ ^]^ (Figure 4B), and delving into connectivity across distinct brain regions^[^ ^123^, ^124^ ^]^ (Figure 4C). The preprocessing of raw EEG data typically involves the use of EEGLAB, along with functions like “eegfilt”, to filter out noise and artifacts. In this process, “eegfilt” is specifically used for band‐pass filtering, which helps in reducing edge artifacts and in obtaining more accurate EEG readings. Following the preprocess, Loreta source localization is generally applied to identify the origins of the electrical activity within the brain, which helps in pinpointing the specific brain regions associated with the recorded electrical signals (Figure 4B). Additionally, the Hilbert transform could extract features from the EEG data, such as the characteristic frequencies of brain waves, instantaneous amplitudes, and phases. The outcomes obtained from EEG data analysis typically include power spectral density plots and topographical maps. The power spectral density plots demonstrate the distribution of signal power across various frequencies, while the topographical maps visually represent the spatial distribution of this power across the scalp. These steps are crucial for analyzing task‐state EEG in the time domain, particularly for identifying event‐related potentials (ERPs), which are distinct waveforms in EEG data that respond to specific stimuli. Conversely, the analysis of resting‐state EEG is predominantly centered on power spectrum analysis to identify variations in power across different frequency bands. Synchrony measures, which utilize coupling functions and simulated histograms, elucidate the regularity and synchronization of neuronal firing, providing a deep understanding of the brain oscillatory dynamics.

Connectivity matrices illustrate the strength and pattern of connections between various brain regions, and phase‐difference estimation highlights the phase relationships between EEG signals, offering insights into the timing of information transfer across these regions (Figure 4C). Connectivity analysis in EEG typically includes both undirected and directed connections. For undirected connectivity, measures such as coherence, phase lock value (PLV), and mutual information are utilized. These assess the degree of synchronization or shared information between different brain regions without specifying the direction of information flow. Directed connections, which provide insights into the directionality of information flow within the brain neural network, are analyzed using methods like the phase slope index and Granger causality‐based indicators. The Mean Vector Length Modulation Index (MVL‐MI) is noteworthy within the broad connectivity framework for understanding complex neural interactions. It quantifies the coupling between the phase of low‐frequency oscillations and the amplitude of high‐frequency activity, thus offering a metric for amplitude‐phase coupling. Similarly, the PLV is used for measuring phase synchronization between EEG signals from different brain regions, revealing the coherence of neuronal oscillatory activities. Furthermore, classification recognition is utilized to interpret these data patterns and classify different brain states or responses, which is vital for applying EEG in diagnostic and monitoring scenarios, where accurate interpretation of brain activity is essential.

Status X condition represents an EEG analytical framework, where interactions or combinations of various statuses such as different conditions, groups, or states of subjects, are mapped against specific conditions including experimental manipulations, environmental factors, or task conditions. This framework usually arises from statistical analysis or computational modeling and aims to elucidate how different brain states are modulated under varying conditions. In this process, matrices are formed that might display the strength of EEG signals, connectivity measures, or statistical outputs from regression analyses. Analyzing these matrix patterns is crucial for understanding how different conditions differentially impact various statuses, thereby offering insights into the complex correlations between brain activity and behavior. In contrast, phase‐difference connectivity (PDC) is employed to assess the directional flow of information between different brain regions. It plays a key role in unraveling the pathways of communication within the brain during various tasks, states, or in response to stimuli. PDC, therefore, is instrumental in providing a comprehensive view of brain dynamics by combining the effects of specific stimuli or conditions with the intricate network of neural interactions in the brain.

Diverse wave patterns detected by EEG are key to uncovering the nuances of neural dynamics. For instance, the alpha band (7−14 Hz), prevalent during relaxation with closed eyes, plays a pivotal role in treatments like vagus nerve stimulation (VNS). Shifts in alpha rhythms during VNS can reflect the effectiveness of epilepsy and depression treatments.^[^ ^123^, ^125^, ^126^, ^127^, ^128^, ^129^ ^]^ On the other hand, the beta frequencies (15−30 Hz) represent alert cognitive states and intersect with attention‐requiring tasks. ERPs, notably the P300 signal that emerges approximately 300 ms post‐visual stimulus, hold significant BMI implications.^[^ ^130^, ^131^ ^]^ Furthermore, theta waves (4−7 Hz) often indicate drowsiness or meditation and correlate with learning and memory, while gamma (30−100 Hz) resonates with high‐order cognitive tasks and information solidification.^[^ ^132^, ^133^ ^]^

Within the sprawling blueprint of applications beyond healthcare, EEG has the potential to improve traffic safety by detecting driver fatigue and issuing timely alerts for rest breaks, thus preventing accidents and reducing traffic congestion.^[^ ^97^, ^134^, ^135^ ^]^ Urban zones could be tailored to promote relaxation by analyzing alpha activity, while areas designated for alert interactions could utilize beta frequencies. Analyzing collective neural responses with different frequency band activities can refine urban design.^[^ ^136^ ^]^ Within this framework, time‐frequency spectra, event‐related spectral perturbations, and scalp maps^[^ ^137^, ^138^, ^139^, ^140^ ^]^ serve as powerful methods to decode intricate brain dynamics. The prevailing appeal of EEG is its non‐invasive nature, whose potential spans from aiding paralyzed individuals to control wheelchairs to enabling computer interactions. Furthermore, advances in wearable EEG, such as dry and capacitive in‐ear electrodes with integrated circuits,^[^ ^141^, ^142^ ^]^ herald a future where BCI is accessible and portable.

### 2.5 ECoG: Monitoring and modulation in practical implementations

ECoG, with its high‐resolution and high‐density electrode arrays spaced mere hundreds of microns apart, offers enhanced signal detection and a broad frequency spectrum, significantly reducing noise compared to EEG (Table 1). This precision enables applications like speech prosthetics,^[^ ^143^ ^]^ which convert ECoG signals from speech articulation into reproduced speech. Unlike EEG, ECoG requires surgical implantation of electrodes either above or beneath the dura mater, directly under the skull, capturing localized and high‐quality signals^[^ ^144^, ^145^, ^146^, ^147^ ^]^ (Figure 4D). This subdural placement is effective in detecting high‐frequency oscillations, especially within the gamma‐band range of 30−150 Hz. Additionally, the modulation by rhythm phase in these activities is promising for applications such as intraoperative cortical mapping^[^ ^148^ ^]^ (Figure 4D). The intricacies of ECoG signals are highlighted by the contrast between high‐frequency (>40 Hz) and low‐frequency oscillations (<40 Hz). Low‐frequency oscillations are notable for their large amplitude and long propagation distance. Their phase could reflect brain connections and is instrumental in the mechanism of information communication across brain regions. These low‐frequency oscillations, associated with states of consciousness and relaxation, can be adjusted by external interventions. In contrast, high‐frequency oscillations, while characterized by low amplitude and short propagation distance, represent different brain activities. These oscillations, particularly in the high‐frequency gamma range, are associated with the activation of local brain areas. The power of these high‐frequency waves is inversely related to their amplitude and is crucial in understanding local brain dynamics. High‐frequency oscillations are typically linked to complex cognitive functions like attention, memory, sensory perception, and inter‐brain region connectivity. Generally, high‐frequency brain signals, unlike low‐frequency oscillations, are inherently generated internally and are less susceptible to external modulation, serving mainly as indicators of various cognitive states.

**TABLE 1:** Comparison and summary of different neural interfaces in the brain for smart city applications.

	EEG	ECoG	MEA^a^	DBS
Electrode location	Scalp	Cortical surface	Subcortical regions	Deep brain
Main utilized signal frequency	Alpha (8−13 Hz) for relaxation and treatment efficacy^b^ like vagus nerve stimulation; beta (13−30 Hz) for alertness and active thought	Low‐frequency (1−4 Hz) for sleep state analysis; high‐frequency (140−165 Hz) for specialized modeling; and gamma frequencies (30−150 Hz) in specific cortical regions for cognitive analysis or sensory perception	Local field potential (<200 Hz) and neuronal spikes (0.1−7 kHz)	High‐frequency stimulation (>70 Hz) for targeted modulation; closed‐loop control typically in beta range (13−30 Hz)
Potential applications	Real‐time feedback, neurofeedback therapy, sleep monitoring, treatment evaluation (EEG synchronization)^c^, connectivity analysis, traffic safety, self‐driving vehicles, cognitive enhancement	Sleep monitoring and enhancement, smart homes, self‐driving vehicles, direct brain‐machine communication, movement prediction, mood/text/voice decoding, epilepsy management and neural disease detection	Neuroprosthetics, cognitive modeling, mood/text/voice decoding, movement prediction, drug delivery, and neural rehabilitation	Motor/limb control, fMRI imaging, mood enhancement, treatments for neurological conditions, robotics and assistive devices, connectivity analyses, cognitive therapies, cognitive learning, mobility treatment, and rehabilitation
Advantages	Non‐invasive and real‐time monitoring	High spatial resolution and localized neural activity	High spatial and temporal resolution, recording and stimulation, compactness (Utah), and adjustable features (Michigan)	Targeted modulation of specific brain regions
Disadvantages	Limited spatial resolution, noise susceptibility	Semi‐invasive procedure, risks associated with brain surface placement	Invasive, surgical implantation risks, bio‐compatibility concerns, and potential limitations with high‐frequency synaptic transmission	Invasive, surgical implantation and stimulation‐related risks

^a^ MEA refers specifically to subcortical microelectrode arrays in this work, while cortical surface electrodes are termed ECoG;

^b^ Anomalies in alpha wave patterns can serve as indicators for diagnosing neurological disorders or identifying cognitive variations;

^c^ Coordinated oscillations of electrical activity in different brain regions, often indicate effective communication between those areas or a specific cognitive state.

In future intelligent cities or smart homes, monitoring high‐frequency gamma activity, linked to cognitive functions like attention and memory, could indicate when residents are deeply engaged in mental tasks, prompting the system to optimize the environment for concentration by adjusting lighting, temperature, and reducing distractions. Low‐frequency oscillations, associated with relaxation or consciousness, could be utilized by smart home systems to induce relaxation or alertness. For instance, in the evenings, the system could enhance relaxation through environmental adjustments like dimming lights, and playing soothing music, thereby enhancing sleep quality. During mornings or when increased alertness is necessary, the environment could be adjusted to energize the residents, perhaps through changes in lighting or ambient sound. This integration of ECoG in smart homes goes beyond simple task automation and energy efficiency,^[^ ^149^ ^]^ aiming to develop living environments that align with the mental and emotional states of residents, potentially transforming how we interact with our surroundings. Additionally, ECoG is crucial in sleep monitoring, enabling a deep understanding of sleep disorders.^[^ ^145^, ^150^ ^]^ By analyzing functional connectivity matrices across frequency bands, such as 1−4 and 140−165 Hz, predictive models for sleep state transitions can be developed.^[^ ^151^ ^]^ Advanced prediction models, such as long short‐term memory (LSTM) units, exhibited high precision, particularly in brain regions affecting sleep like the medial mammillary nucleus and the ventral thalamus.^[^ ^145^, ^152^ ^]^ Complementing this, blood oxygen level‐dependent (BOLD) signals in functional magnetic resonance imaging (fMRI) provide holistic brain dynamics during sleep transitions^[^ ^145^, ^153^, ^154^ ^]^ (Figure 4D). Furthermore, integrating ECoG into autonomous driving technologies could facilitate transitions between manual and automated driving modes (Figure 4D), ultimately reducing traffic congestion.

Leveraging technologies like fMRI and ECoG gamma activity markers can usher in a new era of applications in healthcare, emotion detection, urban safety, and smart home automation. Short‐term ECoG applications, particularly those focused on diagnosing and managing seizures, have been approved. However, the long‐term safety of ECoG for BMI applications remains under investigation. The transition of ECoG from research to real‐world applications requires cooperation involving industrial production, comprehensive clinical trials, and rigorous regulatory oversight.

### 2.6 MEA: Recording and neural modulation in practical implementations

Originating from a single‐electrode system akin to the patch clamp for monitoring bioelectric activity in neurons, MEA has evolved into devices capable of simultaneous recordings from multiple electrode arrays^[^ ^155^, ^156^ ^]^ (Figure 4E). Traditional silicon‐based MEA, notably the Utah and Michigan electrodes, have impacted neurophysiology in the past few decades owing to their high spatial and temporal resolutions. Utah arrays excel in their compactness, while the Michigan electrodes stand out with their adjustable features, adept at capturing signals across varying depths and ranges (Figure 4E). Due to their invasive nature, these tools are essential for capturing detailed electrophysiological signals, including local field potential (LFP) typically below 200 Hz, reflecting collective synaptic potential from neuron groups, and neuronal spikes (0.1−7 kHz), offering insights into individual neuronal activities.^[^ ^157^ ^]^ The integration of both spiking activity and LFP into future BMI promises the creation of dexterous prostheses, paving the way for complex tasks like reach, grasp, and intricate finger movements to become commonplace. By leveraging data from MEA, urban spaces can potentially evolve to be adaptive to inhabitants. This transformative potential opens pathways for groundbreaking enhancements in healthcare, notably in precisely targeted drug delivery systems, personalized neural rehabilitation programs, and the development of neural interfaces that make prosthetic limbs natural and intuitive.^[^ ^158^, ^159^ ^]^

### 2.7 DBS: Stimulation and neural modulation in practical implementations

DBS which targets specific neurons, is generally used for the treatment of neurological conditions like Parkinson's disease.^[^ ^160^, ^161^, ^162^, ^163^ ^]^ Assisted by neuroimaging and targeting techniques, DBS is refining its spatial precision and increasingly focusing on temporal sequencing to enhance treatment efficacy.^[^ ^164^, ^165^ ^]^ Additionally, emerging DBS technologies align with IoT and virtual reality trends by employing encrypted telemetry for wireless data transfer and cloud‐based controls, which extend their uses in real‐world applications beyond healthcare.^[^ ^166^ ^]^

#### 2.7.1 DBS therapy

DBS, employing high‐frequency stimulation typically over 70 Hz, is precisely targeted to specific brain regions like the subthalamic nucleus, commonly associated with Parkinson's disease treatment.^[^ ^165^ ^]^ DBS operates on multiple scales to modulate neural activity, spanning from molecular interactions to broad neuronal network dynamics. On a molecular level, the implanted DBS electrode generates an electrical field that influences voltage‐sensitive sodium channels in neuronal membranes^[^ ^167^ ^]^ (Figure 4F). This stimulation leads to the opening of these channels and the propagation of action potentials along axons. Despite facing challenges such as limited synaptic transmission with high‐frequency signals, DBS effectively serves as a synaptic filter, preventing the spread of abnormal or pathological neural activity, particularly within sensory and motor regions. At the broad neuronal network level, the efficacy of DBS emerges in its modulation of specific neural circuits. For instance, while the thalamus receives inputs from the basal ganglia, it preferentially transmits only those that synchronize with the high‐frequency signals produced by DBS.^[^ ^167^, ^168^ ^]^ This selectivity allows DBS to suppress low‐frequency oscillations without causing widespread network disruption, thereby minimizing its impact on neural plasticity and alleviating symptoms such as akinesia, rigidity, tremor, and dystonia.^[^ ^169^ ^]^

#### 2.7.2 Closed‐loop DBS control

An open‐loop DBS system delivers electrical stimulation without feedback or adjustments based on the outcome. In contrast, a closed‐loop system continuously monitors outcomes to adaptively modify the control action (Figure 4G), thereby enhancing effectiveness and reducing side effects.^[^ ^170^, ^171^, ^172^, ^173^ ^]^ The closed‐loop approach initially focuses on passive sensing and identifying specific neural biomarkers. For instance, upon identifying the gamma biomarker in the amygdala, which is marked by neural activities within the gamma frequency range—often measured via EEG or LFP and indicative of conditions such as anxiety and depression, the closed‐loop system activates stimulation. Within the realm of neural signals, LFP in the beta range (13−30 Hz) stands as an emblem of rigidity and bradykinesia. Conversely, gamma‐band oscillations, particularly from cortical strip electrodes are indicative of dyskinesia. In closed‐loop DBS, two control approaches are prominent^[^ ^90^ ^]^ (Figure 4G). The first utilizes the DBS electrodes for both sensing and stimulation, relying on rhythmic neural signals in either the gamma or beta range to guide stimulation intensity. The second employs external sensors to monitor disease symptoms, which are then fed back to the implanted stimulator to adjust the stimulation timing.

The efficacy of DBS is influenced by several factors, including the frequency and intensity of stimulation as well as the inherent physiological and anatomical characteristics of the targeted region.^[^ ^174^ ^]^ For instance, utilizing low‐frequency DBS below 30 Hz can increase beta oscillations in the subthalamic nucleus. In contrast, beta oscillations, especially within the 13−30 Hz range,^[^ ^175^ ^]^ can disrupt normal neural communication, resulting in behavioral anomalies. These oscillations have gained significant attention as a metric for assessing the clinical condition of patients. Through closed‐loop control typically in the beta range, adjusting the amplitude of the LFP signal can enhance the effectiveness of DBS treatments compared to conventional methods. Moreover, the integration of DBS with IoT technologies heralds a new era in real‐time monitoring, allowing for fine‐tuning treatments based on extensive sensor data. Such a data‐centric approach not only improves DBS efficacy but also paves the way for more timely and adaptive care tailored to individual needs and the diverse applications of closed‐loop controls. The capacity of DBS to precisely modulate neural dynamics—the temporal patterns of neural signaling and connections—opens intriguing possibilities for practical implementations, including adaptive public services and personalized urban experiences that can potentially respond in real time to individual cognitive and emotional states.

#### 2.7.3 DBS frequency modulation and MRI imaging

**FIGURE 5:**
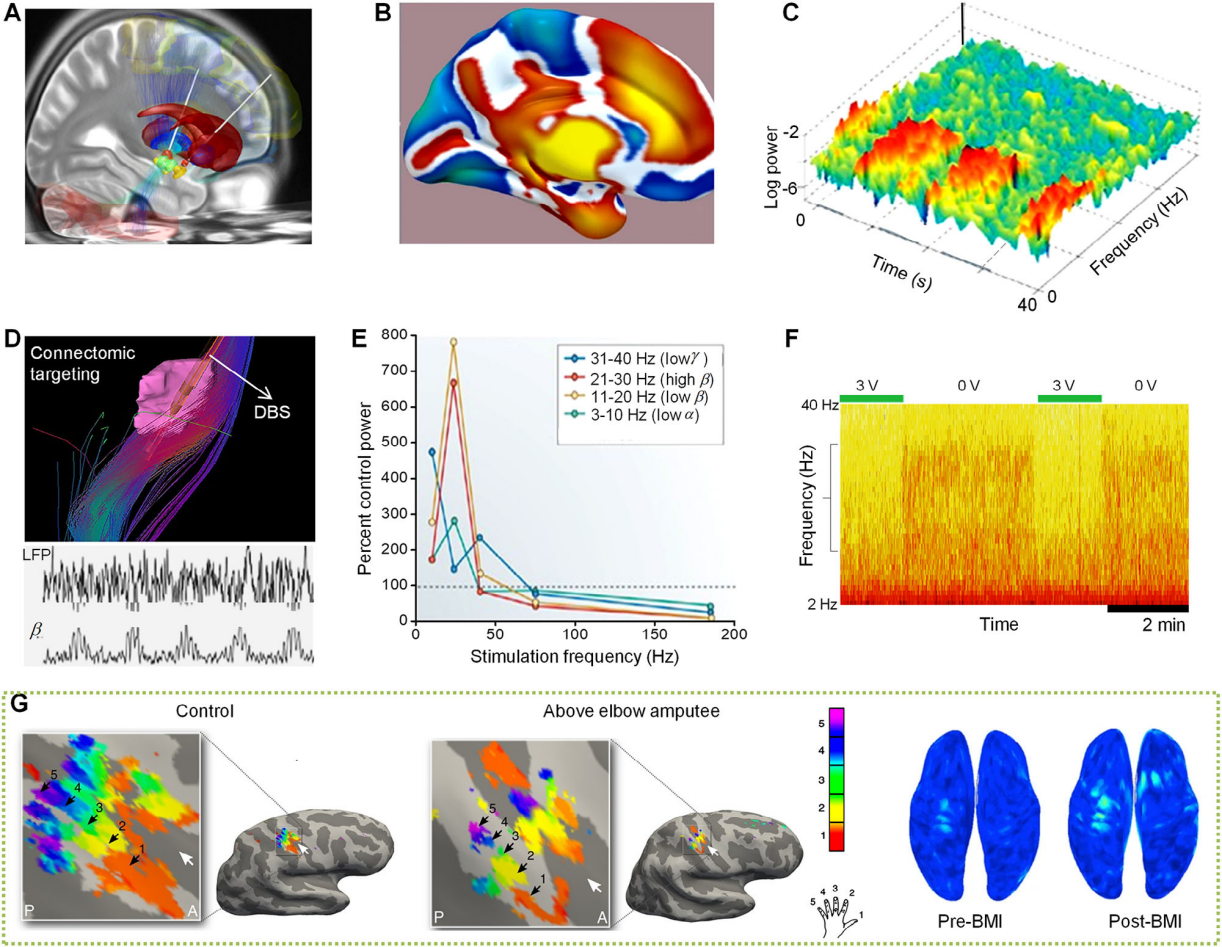
DBS frequency modulation and MRI imaging. (A) DBS electrodes into the striatum (red). Reproduced under the terms of the Creative Commons' Attribution‐Share 4.0 International License. Copyright, The Authors. (B) DBS connectivity. Reproduced with permission.^[^ ^179^ ^]^ Copyright 2017, Wiley‐VCH. (C) Time‐frequency representation of LFP during high‐frequency DBS of ipsilateral subthalamic nucleus. Reproduced with permission.^[^ ^174^ ^]^ Copyright 2007, Springer Nature. (D) DBS connectomic targeting of the ventral nucleus (pink) and beta oscillations. Reproduced with permission.^[^ ^175^, ^176^ ^]^ Copyright 2022, Frontiers; and Copyright 2020, IOP Publishing. (E) Low‐frequency DBS (<30 Hz) enhancing LFP power. Reproduced with permission.^[^ ^62^ ^]^ Copyright 2019, Springer Nature. (F) DBS reducing beta‐band activity in the subthalamic nucleus. Reproduced with permission.^[^ ^68^ ^]^ Copyright 2007, Springer Nature. (G) Ultra‐high‐field MRI for pain sensations imaging: Scans of amputees revealed activation of neurons associated with the movement of missing digits; and sensorimotor plasticity by BMI training for pre‐ and post‐BMI comparison. Reproduced under the terms of the CC‐BY Creative Commons Attribution 4.0 International license (https://creativecommons.org/licenses/by/4.0).^[^ ^181^, ^183^ ^]^ Copyright 2016, The Authors, published by Elife and Springer Nature.

DBS is primarily used for its therapeutic effects on movement disorders, requiring accurate targeting of specific brain areas. In its initial phases, structural MRI is critical for identifying neuroanatomical landmarks. This targeting is further refined through fMRI, essential for ensuring precise and effective modulation of DBS frequencies. Figure 5A illustrates the integration of neural activation models with neuroimaging methods like diffusion tensor imaging, fMRI, and connectomic targeting imaging to optimize DBS‐induced signal variations,^[^ ^176^, ^177^, ^178^ ^]^ thereby facilitating predictions of treatment responses in movement disorders.

Despite its promise, this method faces challenges such as extended durations of fMRI scans and a need for specialized analytical skills, which currently limit its prevalence in current clinical settings. Topographical visualization of neural activity in the brain, which highlights regions involved in specific functions^[^ ^179^ ^]^ (Figure 5B), is crucial for precise electrode placement in DBS. This spatial mapping, correlating brain functions with exact anatomical locations, ensures targeted electrical stimulation of neural areas, essential for effectively treating movement disorders by modulating dysfunctional neural circuits. Figure 5C illustrates the variations in neural oscillations within the beta frequency band over time and frequency.^[^ ^174^ ^]^ These beta oscillations, closely associated with motor control, can be altered through DBS to improve motor function in conditions like Parkinson's disease. Spectrograms serve as crucial tools for clinicians to visualize these oscillations and make informed decisions about the most effective DBS settings for individual patients. By adjusting the stimulation parameters in response to the unique patterns of neural activity observed in the spectrogram, the treatment can be personalized to optimize therapeutic outcomes for individual patients.

Connectomic targeting imaging combines neural activation with neuroimaging, improving electrode placement precision^[^ ^176^, ^178^ ^]^ (Figure 5D). Notably, DBS at 3 V shows significant shifts in beta oscillations^[^ ^175^ ^]^ (Figure 5D), which could offer insights into Parkinson's disease management. Modulating the power of LFPs across different DBS frequencies reveals complex neural response patterns, providing a detailed view of brain electrical activity^[^ ^174^ ^]^ (Figure 5E). LFP modulation indicates the brain immediate response to stimulation and sheds light on the mechanisms by which DBS exerts its effects. By adjusting DBS frequencies and observing the resultant LFP power changes, clinicians can more accurately target therapeutic interventions to the neural basis of movement disorders. Reversible beta oscillation shifts in response to DBS further highlight its potential to tailor neural activities^[^ ^167^ ^]^ (Figure 5F).

High‐field MRI, particularly at 3 Tesla, is the standard in clinical imaging for its high‐resolution capabilities, while ultra‐high‐field MRI at 7 Tesla is primarily a research tool that offers detailed brain structure and function images.^[^ ^180^ ^]^ These imaging modalities, especially when combined with fMRI, provide invaluable insights into brain regions activated by DBS, enhancing our understanding and management of movement disorders and sensory deficits. For instance, ultra‐high‐field MRI imaging captures the activation patterns in amputees, such as the movement of missing digits, shedding light on the neuronal basis of phantom limb pain and the potential for sensorimotor plasticity through BMI training^[^ ^181^, ^182^ ^]^ (Figure 5G). Furthermore, the digit topography revealed by this imaging, characterized by inter‐digit overlaps and digit selectivity, influences tactile interface design for practical applications beyond healthcare,^[^ ^183^, ^184^ ^]^ particularly in developing sensory applications in smart cities.

## 3 NEURAL DECODING

Neural decoding acts as a computational link between the human brain and external devices, employing algorithms and high‐density electrodes to interpret neural signals.^[^ ^185^, ^186^ ^]^ Beyond command recognition, neural decoding has the potential to adapt urban environments according to the emotional and cognitive states of their inhabitants. Envision public information kiosks translating brain signals directly into text or voice, facilitating immediate, hands‐free access to vital information.

### 3.1 Optimized neural decoding algorithms

**FIGURE 6:**
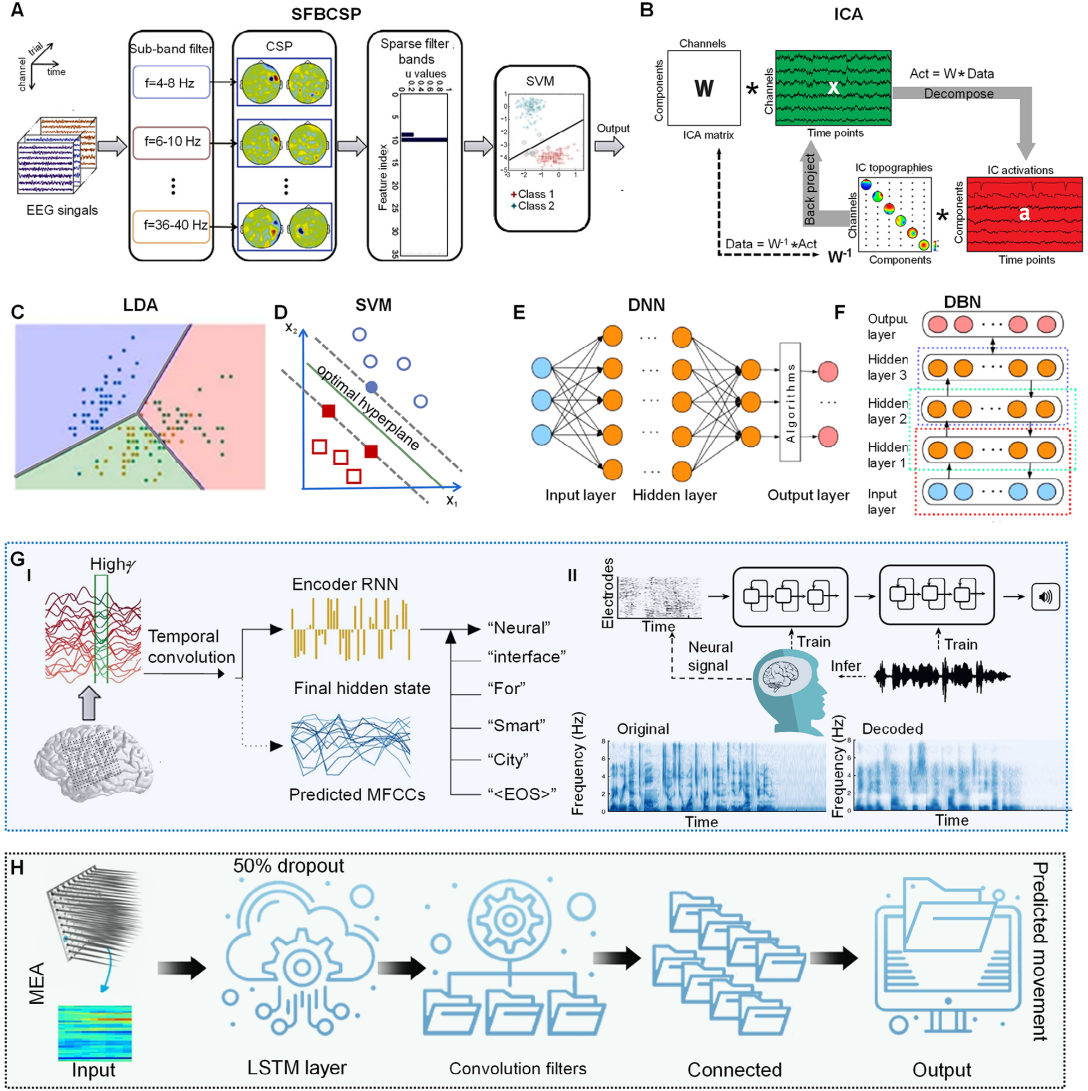
Optimized neural decoding. (A) Sparse FBCSP algorithm for motor‐related action recognition. Reproduced with permission.^[^ ^190^ ^]^ Copyright 2015, Elsevier. (B) ICA of unmixing EEG channels to identify independent components. Reproduced with permission.^[^ ^192^ ^]^ Copyright 2011, Oxford University Press. (C, D) Typical optimized decision boundaries to differentiate between the various classifiers. (C) LDA, and (D) SVM. Reproduced with permission.^[^ ^195^ ^]^ Copyright 2020, MDPI. (E, F) Typical machine learning. (E) DNN and (F) DBN. Reproduced with permission.^[^ ^199^ ^]^ Copyright 2020, Elsevier. (G) Representative ECoG decoding of text (I), reproduced with permission.^[^ ^204^ ^]^ Copyright 2020, Springer Nature; and voice (II), reproduced with permission.^[^ ^205^ ^]^ Copyright 2019, Springer Nature. (H) Representative MEA decoding for movement prediction. LSTM, long short‐term memory layer. Reproduced with permission.^[^ ^209^ ^]^ Copyright 2018, Springer Nature.

Advancements in neuroscience and machine learning have led to optimized neural decoding, facilitating efficient communication between devices and the brain.^[^ ^187^ ^]^ For individuals suffering from degenerative motor diseases, the decoding of brain neural signals is essential, as it transforms neural signals into understandable outputs.^[^ ^185^, ^188^ ^]^ The Filter Band Common Spatial Pattern (FBCSP) method, an enhancement over the standard CSP employs a filter bank to obtain features across multiple frequency bands^[^ ^189^, ^190^ ^]^ (Figure 6A). This leads to improved BCI accuracy and has applications ranging from neurorehabilitation to immersive gaming and virtual reality experiences.^[^ ^191^ ^]^ ICA is another technique that enhances EEG or ECoG signal decoding by separating pure signals from noise^[^ ^192^, ^193^ ^]^ (Figure 6B). It increases the signal‐to‐noise ratio, preserving valuable data while excluding disturbances. By isolating statistically independent cortical processes, ICA finds applications in neurology, cognitive neuroscience, and neuroengineering.^[^ ^194^ ^]^ BCI systems that use classifiers such as artificial neural networks (ANN) or linear discriminant analysis (LDA)^[^ ^195^, ^196^ ^]^ can control humanoid robots through brain signals (Figure 6C). Integration with inputs from multiple sensors opens possibilities in rehabilitation, communication, and device control. Support vector machine (SVM) algorithms, known for their noise resilience and efficient data handling,^[^ ^195^, ^197^, ^198^ ^]^ are instrumental in directing prosthetic devices and aiding individuals with motor and communication difficulties (Figure 6D). Furthermore, deep learning algorithms, including LSTM, deep neural networks (DNN), deep belief networks (DBN), and convolutional neural networks (CNN), are valuable in BCI applications^[^ ^199^, ^200^, ^201^, ^202^, ^203^ ^]^ (Figure 6E,F) due to their role in feature identification and signal decoding, but require careful optimization to prevent overfitting and simplify their complex learning procedures. The evolution of these algorithms not only empowers individuals with enhanced capabilities but also reshapes urban experiences toward inclusivity and cutting‐edge technological integration.

### 3.2 Text, voice, and movement decoding

Algorithms such as recurrent neural networks (RNN) and LSTM have significantly enhanced our ability to decode neural signals.^[^ ^200^, ^201^ ^]^ These computational tools are instrumental in translating brain signals into actionable data, extending their applications beyond the traditional medical domain. One pivotal study demonstrated the capability of RNN decoding to transform ECoG signals into textual representations^[^ ^204^ ^]^ (Figure 6G). Upon comprehensive model training, this approach has applications not just in medical devices like speech aids but also within the broad IoT framework of smart city infrastructures, facilitating an environment where citizens can seamlessly interact with integrated intelligent systems. Moreover, recent advancements have enabled the conversion of brain activity directly into audible speech^[^ ^205^ ^]^ (Figure 6G). This holds promise for individuals who are speech‐impaired due to neurological conditions, and it opens the door for voice‐activated functionalities. Reflecting on the history of BCIs, these interfaces were originally developed to reinstate communication abilities in individuals with significant disabilities.^[^ ^206^ ^]^ A notable example is the P300 speller, developed to enable patients to type text on a computer screen utilizing brain activities.^[^ ^207^ ^]^ Paired with speech synthesizers, these technologies have evolved to potentially decode full sentences from minimally invasive brain recordings, suggesting a future of prostheses for speech restoration and privacy‐respectful communication forms like silent‐speech interfaces.^[^ ^208^ ^]^

In addition, strides have been made in developing an MEA‐based system specifically for movement decoding^[^ ^209^, ^210^ ^]^ (Figure 6H). This system can translate neural signals into accurate movement predictions. Offering benefits ranging from assisting people with motor disabilities to enhancing interactions with robotic assistive systems in urban settings. Various machine learning algorithms, such as regression models, linear classifiers, DNN, and SVM, have been utilized to achieve high‐accuracy discrimination of movement intentions. This includes both broad and precise motor movements, as demonstrated in able‐bodied and paralyzed participants using ECoG electrodes and Utah arrays.^[^ ^211^ ^]^

### 3.3 Cognitive therapies and treatments

In recent years, BCIs have gained prominence as potential therapeutic tools for neuropsychiatric conditions like depression and anxiety.^[^ ^212^ ^]^ A major challenge lies in accurately decoding mood states, which is crucial for both diagnosis and treatment. Recent strides in closed‐loop DBS treatments have been pivotal in addressing this challenge. Further, the integration of wirelessly transmitted BCIs into smart city infrastructures offers promising avenues for mood decoding, stress reduction through mindfulness, and cognitive learning and training.

#### 3.3.1 Mood decoding

**FIGURE 7:**
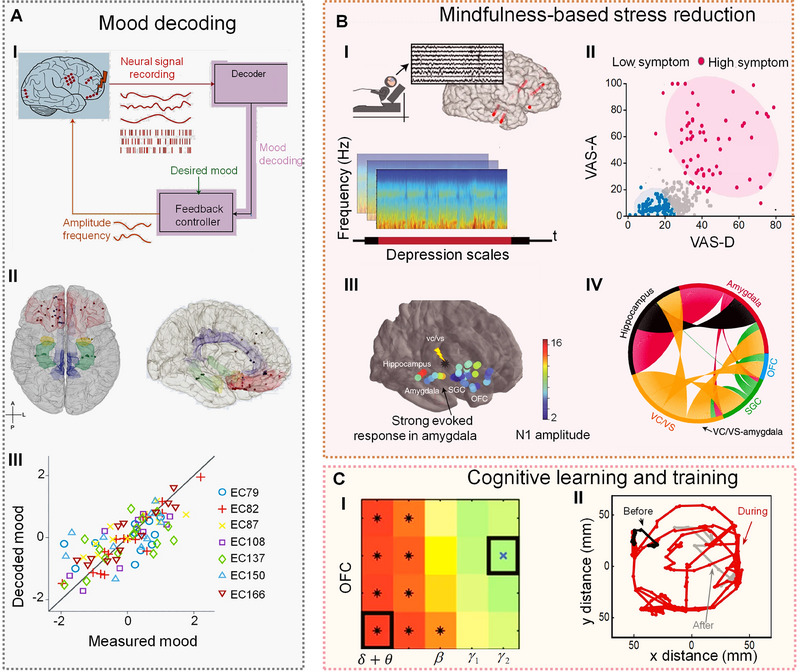
Neural decoding. (A) Mood decoding. Reprinted with permission.^[^ ^39^ ^]^ Copyright 2019, Springer Nature. (I) Closed‐loop electrical stimulation for therapies and feedback; (II) multisite neural activity mapping via dynamic latent state‐space models; (III) decoded mood relation. (B) Neural decoding for mindfulness stress reduction. Reprinted with permission.^[^ ^170^ ^]^ Copyright 2021, Springer Nature. (I) Intracranial electrodes for biomarker identification; (II) two symptoms for depression and anxiety; (III) evoked potentials in the corticolimbic network; (IV) hemisphere network with circumference strength and color‐coded start locations. (C) Neural decoding for cognitive learning and training. (I) Spectral‐geometric emotion‐forecasting neural pathways (green for positive and red for negative correlations), reproduced with permission.^[^ ^223^ ^]^ Copyright 2018, Springer Nature; (II) the trajectory before (black), during (red), and after (grey) near‐infrared DBS stimulation, reproduced with permission.^[^ ^224^ ^]^ Copyright 2022, Springer Nature.

Accurate mood decoding is crucial for the effective treatment of mood disorders, but real‐time tracking of emotional states remains a significant challenge due to the complex interactions within neural systems.^[^ ^213^, ^214^ ^]^ Advances in neuroimaging provide insights into the neural basis of emotional responses, yet the intricate dynamics within the cortical and limbic systems require further exploration. Closed‐loop DBS offers a solution by enabling real‐time mood state decoding and facilitating targeted electrical therapies^[^ ^212^, ^215^ ^]^ (Figure 7A). Unlike motor‐function BCIs, which often employ algorithms like FBCSP and SVM, mood BCIs use closed‐loop systems with both control and stimulation components, leveraging machine learning algorithms, such as DNN or LSTM (Table S1), to analyze neural activity in various brain regions and identify specific mood states^[^ ^39^, ^216^, ^217^ ^]^ (Figure 7A). Combining feedback controllers for mood‐based stimulation adjustment with neural decoders for mood identification enables the potential for personalized treatments. Key neural regions like the limbic system and the orbitofrontal cortex play crucial roles in this decoding process. Integrating advances in mood research with urban planning, mood BCIs can reshape city designs to be attuned to residents' emotional states, elevating both individual well‐being and the overall urban experience.

#### 3.3.2 BCI‐enhanced mindfulness

Mindfulness practice, known for reducing stress and improving cognitive function, could be further enhanced by integrating BCIs that provide real‐time neural feedback.^[^ ^218^, ^219^ ^]^ Cutting‐edge methods such as implanting electrodes to monitor brain activity^[^ ^170^ ^]^ and targeting specific neural regions associated with major depressive disorder symptoms^[^ ^170^, ^220^ ^]^ present the groundbreaking possibility for highly personalized neurostimulation therapies (Figure 7B). Further insights into emotional processing mechanisms can be garnered by analyzing the N1 amplitude of evoked potentials,^[^ ^170^, ^221^ ^]^ which is the negative voltage peak observed approximately 100 ms after stimulus and is commonly used to investigate attention and sensory processing. Additionally, stimulation‐induced mapping of brain region connections underscores the significant impact of DBS on connectivity (Figure 7B). Urban areas equipped with BCI‐enhanced mindfulness interventions can be tailored to cater to individual needs, thereby providing valuable resources for stress mitigation.

#### 3.3.3 Cognitive learning and training

DBS‐based BCI approaches offer the potential for cognitive learning and training, particularly for individuals with cognitive deficits.^[^ ^222^ ^]^ These methods provide real‐time feedback, enabling individuals to assess and improve their cognitive skills. Analysis across different frequency bands can reveal the temporal predictability of mood states, underscoring the dynamic capabilities of neural encoding models^[^ ^223^ ^]^ (Figure 7C). Passive BCIs, which are part of this technology spectrum, enhance high‐order brain functions like reasoning and decision‐making by monitoring brain activity. This includes assessing decisions‐making processes and confidence levels in those decisions. Another promising development is the use of near‐infrared deep brain modulation in cognitive enhancement, which demonstrates the potential to improve cognitive abilities non‐invasively^[^ ^224^, ^225^, ^226^ ^]^ (Figure 7C). Furthermore, the integration of neurotechnologies in cognitive enhancement is poised to facilitate effective human–AI collaboration. While AI excels in computation‐intensive tasks, like playing Go, humans outperform AI in tasks requiring advanced reasoning and intricate problem‐solving skills. Future neurotechnologies are expected to enhance these human strengths, enhancing performance in a variety of tasks through efficient human‐AI collaboration.

## 4 NEURAL INTERFACES FOR WEARABLE INTERACTIONS

The rapid evolution of neurotechnology has ushered in a new era of wearable interactions, seamlessly integrating the human brain with external devices.^[^ ^171^, ^227^, ^228^, ^229^ ^]^ Central to this progression are wearable BCIs and haptic interactions, which together, revolutionize our connection to urban landscapes and digital platforms. Wearable BCIs, encompassing designs like headsets, EEG‐integrated smart glasses, and baseball caps, provide continuous monitoring of neural activities, promising vast implications in healthcare, sports, and gaming. On the other hand, haptic interactions, tailored for tactile communication, bring a tangible dimension to digital experiences,^[^ ^172^, ^230^ ^]^ ranging from force feedback in virtual reality to intuitive touch in public service kiosks.

### 4.1 Wearable BCIs

**FIGURE 8:**
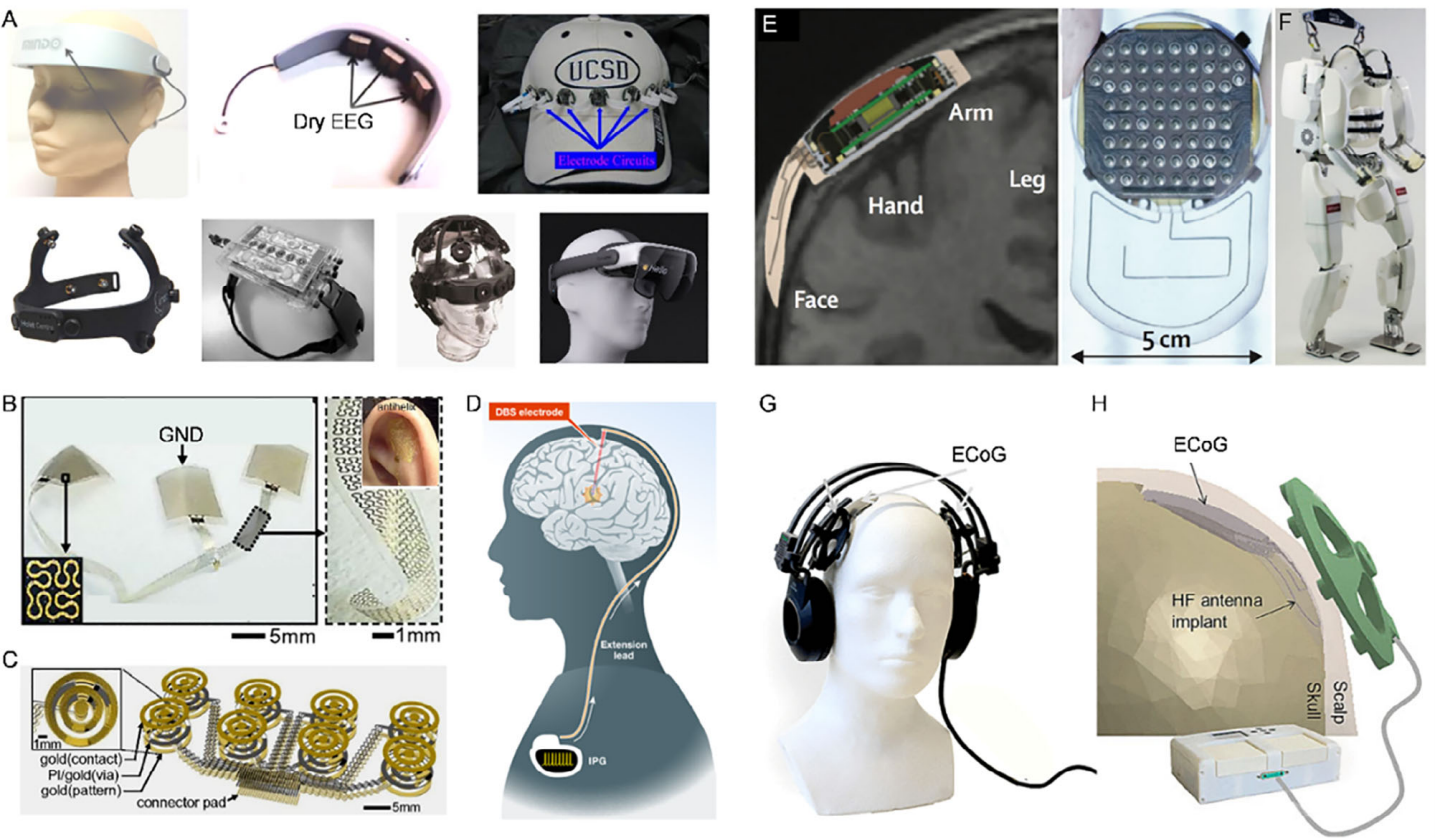
Wearable BCI devices. (A) Wearable EEG devices. Top: headset, reprinted with permission.^[^ ^231^ ^]^ Copyright 2012, Springer; baseball cap, reprinted with permission.^[^ ^233^ ^]^ Copyright 2008, IEEE. Bottom: monitoring headset, reprinted with permission.^[^ ^287^ ^]^ Copyright 2010, IEEE; wireless steady‐state visual evoked potential (SSVEP) device, reprinted with permission.^[^ ^232^ ^]^ Copyright 2006, IEEE; commercial Quasar DSI 10/20, reprinted with permission.^[^ ^288^ ^]^ Copyright 2021, Quasar; and smart glass, reprinted with permission.^[^ ^234^ ^]^ Copyright 2021, Cognixion. (B, C) EEG skin devices including fractal device architectures and tripolar concentric ring and capacitive designs. B,C are reproduced with permission.^[^ ^142^ ^]^ Copyright 2015, National Acad Sciences. (D) DBS clinically implanted device. Reproduced with permission.^[^ ^235^ ^]^ Copyright 2019, Embopress. (E–H) Wearable ECoG devices. (E) WIMAGINE anatomical implant, reproduced with permission.^[^ ^236^ ^]^ Copyright 2014, IEEE; (F) wearable Clinatec device, reproduced with permission.^[^ ^237^ ^]^ Copyright 2019, Clinatec; (G, H) WIMAGINE wired and wireless wearable ECoG devices, reproduced with permission.^[^ ^236^ ^]^ Copyright 2014, IEEE.

Wearable EEGs come in diverse designs like headsets,^[^ ^231^ ^]^ headbands,^[^ ^232^ ^]^ baseball caps,^[^ ^233^ ^]^ and smart glasses^[^ ^234^ ^]^ (Figure 8A). These devices are tailored for comfort and continuous brain activity monitoring. EEG skin devices, featuring mesh electronics with stretchable interconnectors,^[^ ^142^ ^]^ conform to the contour of the skin (Figure 8B,C), offering enhanced resolution and robustness. Wearable DBS devices treat neurological and psychiatric conditions^[^ ^235^ ^]^ (Figure 8D), while the WIMAGINE implant is a wearable ECoG device, replaces part of the cranium to streamline surgery and improve safety^[^ ^236^ ^]^ (Figure 8E). The CLINATEC device, worn on the head, captures ECoG data for interpretive movements^[^ ^237^ ^]^ (Figure 8F), which is currently undergoing clinical trials, showing promise for patients with severe disabilities. The WIMAGINE systems, both in wired and wireless versions, have been utilized in applications such as controlling motorized exoskeletons using brain signals^[^ ^236^ ^]^ (Figure 8G,H). These wearable BCIs promise a transformative impact on urban living, including stress monitoring, telehealth solutions, and controlling robots and transport systems.

### 4.2 Haptic interactions

**FIGURE 9:**
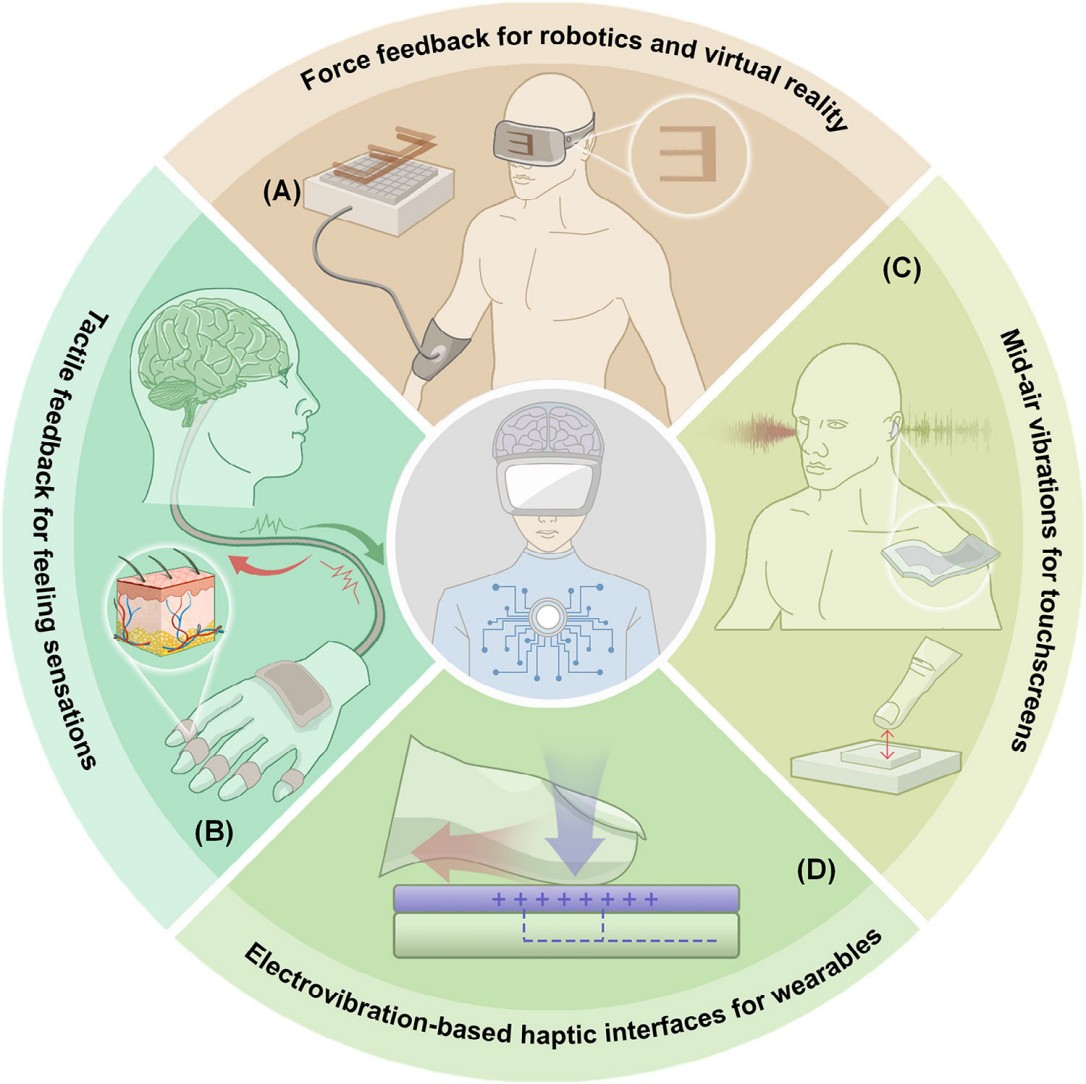
Neural interfaces for haptic interactions. (A) Force feedback for robotics and virtual reality. (B) Tactile feedback technologies, such as triboelectric sensors, designed for feeling sensations. (C) Mid‐air vibrations for touchscreens and similar interfaces, incorporating technologies like ferroelectric, electromagnetic, ultrasonic piezoelectric, and thermoelastic laser devices. (D) Electrovibration‐based haptic interfaces for wearables such as gloves, featuring pneumatic, piezoelectric, thermoelectric, or ferroelectric actuators; and heating haptic interfaces.

Neural interfaces tailored for tactile communication are reshaping our interactions. Force feedback, crucial for virtual reality and robotics, can be enhanced by neural interfaces like EEG or DBS, enabling tactilely immersive experiences and intuitive robotic‐assisted public services (Figure 9A). Tactile feedback technologies, when integrated with neural interfaces like EEG or ECoG, provide direct sensations of touch; these systems simulate finger sensations and optimize interactions using sensors like hydraulically amplified taxels^[^ ^238^, ^239^, ^240^, ^241^ ^]^ (Figure 9B).

Mid‐air haptic technologies provide contactless sensations in the air and, when integrated with neural interfaces, enable public venues to offer air‐touch menus controlled by EEG‐detected neural patterns^[^ ^242^, ^243^, ^244^, ^245^, ^246^, ^247^, ^248^ ^]^ (Figure 9C). Electrovibration technologies, essential for wearable devices, when combined with neural interfaces like EEG or invasive methods such as ECoG and MEA, enable haptic gloves to provide an intuitive touch experience in kiosks and digital installations^[^ ^249^, ^250^ ^]^ (Figure 9D). These wearable devices feature diverse actuators—from pneumatic to piezoelectric, thermoelectric, and ferroelectric—and can include heating interfaces^[^ ^251^, ^252^, ^253^, ^254^, ^255^, ^256^, ^257^ ^]^ to deliver detailed tactile sensations. The convergence of haptic technologies with neural interfaces paves the way for tactile interactivity in practical applications, amplifying accessibility and enriching user experience (Table 2).

**TABLE 2:** Comparison of different haptic interfaces for real‐world applications beyond healthcare.

Haptic devices	Description	Structure	Applications	Ref.
**Electrovibration for haptic gloves and other wearable devices**				
Flexible pneumatic electrovibration glove	Provides realistic experience of virtual objects with tactile feedback	Pneumatic soft actuator, interface board, piezoelectric sensors, and high‐voltage converter	Gaming, educational training, medical training	[249]
Piezoelectric glove	Identify user gestures	Kapton film, AlN layers, and Mo‐based electrode	Healthcare, rehabilitation	[250]
Thermo‐haptic device	Skin‐like mechanical properties	Si nanomembrane diodes, and electrode arrays	Human‐machine interfaces	[252]
Joule heating interfaces	Efficient heat transfer	Ag nanowires, SBS thermoplastic elastomer, serpentine‐mesh structure	Virtual reality, gaming	[253]
Thermal electric actuators	Provide haptic feedback through heat transfer, creating warm or cool sensations	p‐, n‐type Bi_2_Te_3_ pellets, and low thermal conductivity polymer	Virtual reality, gaming, and medical devices	[254]
Thermal electric actuators (audio)	Provide tactile and audible feedback	Ag nanowires, touch sensors, ribbon‐shaped actuators, and electrode grid	Human–computer interaction, gaming, and mobile devices	[255]
**Tactile feedback for feeling sensations**				
Ring‐like tactile device	Multi‐sensory feedback and sensing rings	Triboelectric nanogenerator (TENG) sensors, and NiCr heaters	Sensory devices, and haptic interfaces	[238]
Hydraulically amplified taxels (HAXEL)	Provide flexible and dense cutaneous haptic feedback	Fluid‐filled cavity, non‐stretchable polymer coating, and segmented electrodes	Wearable haptics, microfluidics, soft robotics, and virtual reality	[239]
Piezoelectric glove	Enables multidimensional motion detection and prompt haptic feedback	Piezoelectric stimulators, and elastomer‐derived triboelectric sensors	Virtual reality, robotics, and rehabilitation	[240]
Triboelectrification	Mimics Merkel cells for touch and pressure detection	Receptive substrate, and triboelectric capacitive potential	Prosthetics, smart wearables, and intelligent systems	[241]
**Mid‐air vibration for touchscreens and other user interfaces**				
Ferroelectric‐based dynamic interfacing	Utilizes a ferroelectric layer to transform mechanical energy into electrical energy, and mimics synapses	Ferroelectric layer, receptive substrate, and transistor	Robotics, virtual reality, and haptic feedback systems	[242]
Piezoelectric micromachined ultrasonic transducer (PMUT)	Thin layer of piezoelectric material patterned into an array of dots or squares	Piezoelectric material, and thin metallic layer	Medical imaging, therapeutic ultrasound and cell manipulation	[243, 244]
Ultrasound haptic devices (e.g. Ultraleap)	Ultrasound array creating points of pressure on the skin	Ultrasound arrays	Virtual reality, augmented reality, and haptic feedback	[245, 246]
Indirect laser radiation	Utilizes a laser system and an elastic substance to stimulate the skin through thermoelastic effects	Laser and elastic substance	Virtual reality, gaming, and human‐computer interaction	[247]
Electromagnetic arrays or rotating magnet disks	Small electromagnets or rotating disks generating a magnetic field that induces air vibrations	Electromagnets or rotating disks	Virtual reality, augmented reality, and medical training simulations	[248]

Force feedback for robotics and virtual reality				
Self‐powered electrotactile device	Electrotactile interface combined with a TENG array, inducing a discharge signal to the skin	Electrotactile interface, and TENG arrays	Robotics and virtual reality	[289]
Prosthetic sensory‐motor interfaces	Controlled and powered wirelessly, frequencies between 100 to 300 Hz provide strong sensations	Wireless components, and frequency modulation	Prosthetics, rehabilitation, virtual reality, and augmented reality	[290]
Augmented virtual reality with force feedback	Interconnected virtual environments with haptic feedback delivered through gloves, suits, or exoskeletons	Gloves, suits, and exoskeletons	Robotics and virtual reality	[238]
Force feedback for robotic or limb control	Sensors, control algorithms, and actuators deliver tactile feedback	Sensors, control algorithms, and actuators	Prosthetics, professional training simulators and virtual reality	[291]

## 5 NEURAL INTERFACES FOR AI‐DRIVEN BCI

AI‐driven BCIs that facilitate direct interactions between the external devices and human brain are revolutionizing how we interact with our digital environments.^[^ ^258^ ^]^ This integration promises not only to enhance efficiency but also to provide personalized interaction models tailored to individual needs.^[^ ^259^ ^]^ AI‐driven BCIs can redefine urban living, enabling actions like altering street illumination with a thought, orchestrating traffic flows to mitigate congestion, or even steering autonomous vehicles. Virtual assistants, already a staple in urban life, could evolve to be more responsive and intuitive through BCI integration.^[^ ^260^ ^]^

### 5.1 BCI operation modes in real‐world applications beyond healthcare

AI‐driven BCIs extend their impact beyond healthcare, enabling thought translation into actionable commands for applications like rehabilitation and enhanced communication. Software advancements in machine learning and signal processing have improved the signal‐to‐noise ratio in neural signal recording, potentially allowing non‐invasive BCIs to rival invasive ones in performance while reducing health risks and costs. Open‐source software tools like EEGLAB and OpenViBE have accelerated BCI research by providing accessible signal processing and machine learning. However, hardware development has lagged due to high costs and lengthy development processes. Advancements include biocompatible invasive interfaces and the shift from wet to dry in‐ear EEG electrodes, making brain recording more accessible and efficient.

#### 5.1.1 Communication mode

**FIGURE 10:**
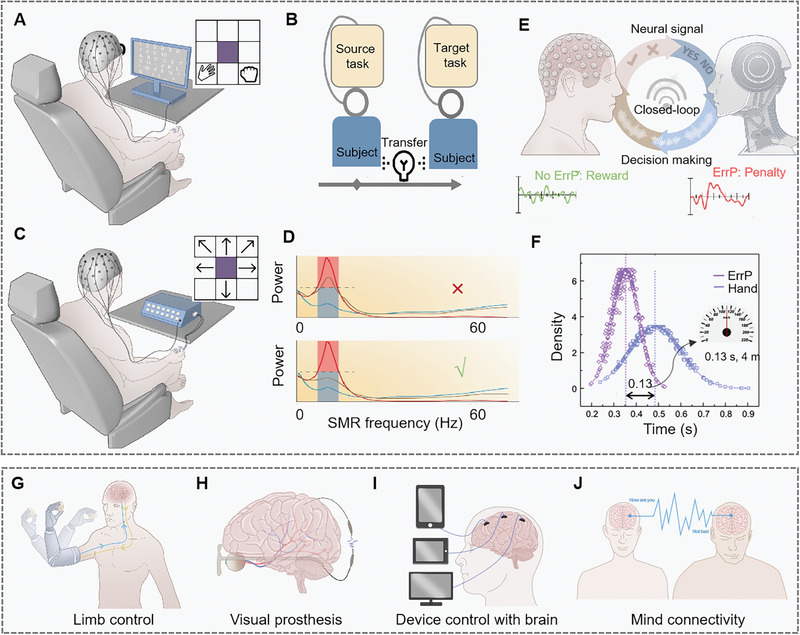
AI‐driven BCIs. (A) Teleoperated communication and mode; (B) BCIs transfer learning; (C) autonomous modes; (D) EEG‐driven motor intention for enhanced instrumental learning, reproduced with permission.^[^ ^272^ ^]^ Copyright 2013, Wiley‐VCH. (E) brain‐AI closed‐loop system; (F) ErrP feedback versus manual stop button in autonomous navigation system. Reproduced with permission.^[^ ^68^ ^]^ Copyright 2022, Springer Nature. Potential applications of (G) limb control; (H) visual prosthesis; (I) remote control; and (J) mind connectivity.

Teleoperated communication mode utilizes EEG‐based BCIs to control external devices, such as wheelchairs and traffic light systems, using neural signals^[^ ^261^ ^]^ (Figure 10A). Neural signals undergo processes of filtration, augmentation, and categorization through algorithms,^[^ ^262^ ^]^ enabling control of devices and influencing direct stimulation of brain areas or muscle groups.^[^ ^263^ ^]^ The primarily non‐invasive nature of this BCI mode allows for diverse smart city applications, including traffic management, air quality control, and energy optimization.^[^ ^264^, ^265^, ^266^, ^267^ ^]^ Another application in this domain is the capacity to recreate tactile feedback using intracortical micro‐stimulation (ICMS), a method that involves using microelectrodes to deliver electrical currents to specific brain cortical regions for mapping neural circuits or inducing artificial sensations or movements.^[^ ^268^ ^]^ This can offer transformative experiences, especially in the realm of prosthetic design. AI techniques, like transfer learning, refine the neural intention decoding process^[^ ^269^ ^]^ (Figure 10B).

#### 5.1.2 Movement mode

Autonomous movement BCIs primarily focus on restoring neural activity in patients with conditions like strokes or spinal cord injuries^[^ ^261^, ^270^, ^271^ ^]^ (Figure 10C). Non‐invasive methods such as EEG are used for guiding devices based on motor intentions^[^ ^272^ ^]^ (Figure 10D), while invasive approaches, involving surgical placement of electrodes like ECoG or MEA, are required for more precise interventions. Neural bypasses and bridges reroute signals around injured nervous system parts, linking decoded signals to electrical muscle or nerve stimulation to potentially restore movement. The first use of a neural bypass for restoring voluntary movement in human involved placing a MEA electrode array on the primary motor cortex.^[^ ^273^ ^]^ This allowed for the deciphering of finger and hand motions, and subsequently, more intricate actions, illustrating its potential in restoring motor function. The potential future uses of this mode include directing robots and vehicles for urban services like deliveries and maintenance. Both teleoperated and autonomous BCIs offer unique advantages for diverse applications. Teleoperated communication BCIs can control traffic, monitor air quality, and regulate energy systems, while autonomous movement BCIs can manage autonomous vehicles or robots. Integrating these two modes could redefine urban services, elevating their efficiency and user accessibility.

### 5.2 Closed‐loop AI‐BCI systems in real‐world applications beyond healthcare

BCI development typically progresses from identifying strong neural patterns in controlled lab experiments to testing these patterns in realistic settings through open‐loop BCIs, which operate without user feedback. The next stage is closing the loop, creating neuroadaptive AI‐BCIs that update in real time based on the user mood state. While open‐loop BCIs have been widely explored, closed‐loop systems are less investigated but offer more seamless user interaction, such as BCIs for arousal regulation in flight simulators and therapeutic BCIs for controlling epileptic seizures and restoring emotional function in neuropsychiatric disorders. Closed‐loop AI‐BCIs, in contrast to one‐way open‐loop systems, are adaptive, utilizing real‐time user feedback for ongoing optimization. Current challenges include synchronizing operations in real‐time, especially using visual evoked potentials (VEPs)^[^ ^274^, ^275^ ^]^—neural responses to visual stimuli to assess the functionality of visual pathways—and ERPs^[^ ^276^ ^]^—brainwaves triggered by specific sensory events. To address these, asynchronous BCIs have been developed.^[^ ^277^ ^]^ These systems operate independently of external cues, enabling continuous, natural interaction between the user and the system, and have achieved advancements like a transmission speed of 67.7 bit/min^[^ ^278^ ^]^ and the ability to detect error‐related potentials (ErrP)—neural signals generated when an error is perceived.^[^ ^68^, ^279^ ^]^ A key example is the BACLoS^[^ ^68^ ^]^ (Figure 10E), which continually refines decision‐making via a feedback loop. BACLoS generally uses EEG devices and low‐power platforms like SpiNNaker, TrueNorth, and Loihi resembling earbuds for high‐quality data capture. ErrP feedback has notably reduced reaction times^[^ ^68^ ^]^ (Figure 10F) in scenarios like driving.

### 5.3 AI‐driven BCIs for future smart city applications

AI‐driven BCIs hold the potential to address urban challenges and pave the way for human‐machine interactions. Applications range from limb and robot control to visual prostheses, enabling remote control and mind connectivity.

#### 5.3.1 Limb, robot control, and clinical applications

Traditional prostheses rely on electromyography (EMG) signals from peripheral muscles, offering limited information about movements like hand opening or closing. BCIs can aid in motor control recovery after stroke or multiple sclerosis by bypassing the impaired neuromotor system. Next‐generation neural prostheses will capture detailed motor intentions directly from brain activity, offering precise control and seamless integration with the body. Despite challenges in speed and control accuracy, advancements in software and hardware, along with hybrid neural interfaces combining multiple signals (e.g. ECoG, MEA, EEG,) or BCI paradigms (e.g. SSVEP and P300), are expected to improve robotic arms control^[^ ^280^ ^]^ (Figure 10G). These BCIs allow robots to conduct complex tasks, such as waste management and surveillance.^[^ ^281^ ^]^ Invasive BCIs are crucial in medical rehabilitation, linking the brain and muscles to restore neural function in limb‐impaired individuals. Non‐invasive EEG‐based BCIs, while more accessible, often provide limited control and depend on AI for enhanced accuracy. Techniques like random forest algorithms improve non‐invasive sensorimotor rhythm BCI accuracy by effectively assessing somatosensory evoked potentials (SEPs)—electrical signals measured via EEG that are generated in response to tactile or proprioceptive stimuli, often used to evaluate the integrity of sensory pathways.^[^ ^282^ ^]^ Research is underway to decode neural signals of somatosensory experiences in healthy individuals, with goals to replicate these stimuli using methods like ICMS,^[^ ^283^ ^]^ potentially enhancing virtual reality and simulation training experiences.

#### 5.3.2 Prostheses and future urban transportation

Visual prostheses, crucial in biomedical engineering, offering hope to those with blindness resulting from conditions such as age‐related maculopathy or congenital amaurosis^[^ ^284^ ^]^ (Figure 10H), are evolving with AI‐driven BCIs, tailoring electrode configurations to individual needs. The shift to a flexible multielectrode system could refine retinal stimulation to align with the intricacies of human retinal physiology. Beyond medical solutions, visual prostheses can enhance urban living, promising for adaptive lighting tailored to individual needs and augmented reality enhancements that turn simple city tours into immersive experiences. In the broad urban landscape, the influence of visual prostheses is also reshaping the future of transportation. These interfaces are poised to revolutionize self‐driving cars by introducing thought‐controlled navigation systems. Such developments could enhance road safety, with drivers guiding vehicles using their thoughts, thus decreasing accidents due to human errors or fatigue.

#### 5.3.3 Remote device control, and smart homes

Neural interfaces integrated with IoT offer transformative potential in the realm of smart devices (Figure 10I), which allow direct thought‐to‐device communication, obviating the need for physical intermediaries. From modulating home settings to plunging into virtual realities, the horizon of possibilities is vast. Specific neural patterns associated with various commands are identified by advanced algorithms, ensuring that a range of IoT devices, including thermostats and lighting systems, are controlled precisely and reliably. Picture a smart home where brain‐controlled devices allow residents unprecedented control over their environment while promoting sustainability and enhanced health outcomes. Advances in nanotechnology and wireless capabilities are anticipated to produce BCIs that are both small in size and compatible with biological tissues.^[^ ^96^, ^285^ ^]^

#### 5.3.4 Communication and shared experiences

In future smart cities, BCIs could go beyond basic control to facilitate sharing emotions and thoughts^[^ ^286^ ^]^ (Figure 10J). This interaction could remove the need for physical interfaces altogether, providing extraordinary accessibility in telemedicine, remote collaborations, and entertainment domains such as thought‐driven gaming. However, challenges in reliability, protecting privacy, and accurately decoding complex brain neural signals remain significant.

## 6 CONCLUSIONS AND PERSPECTIVES

**FIGURE 11:**
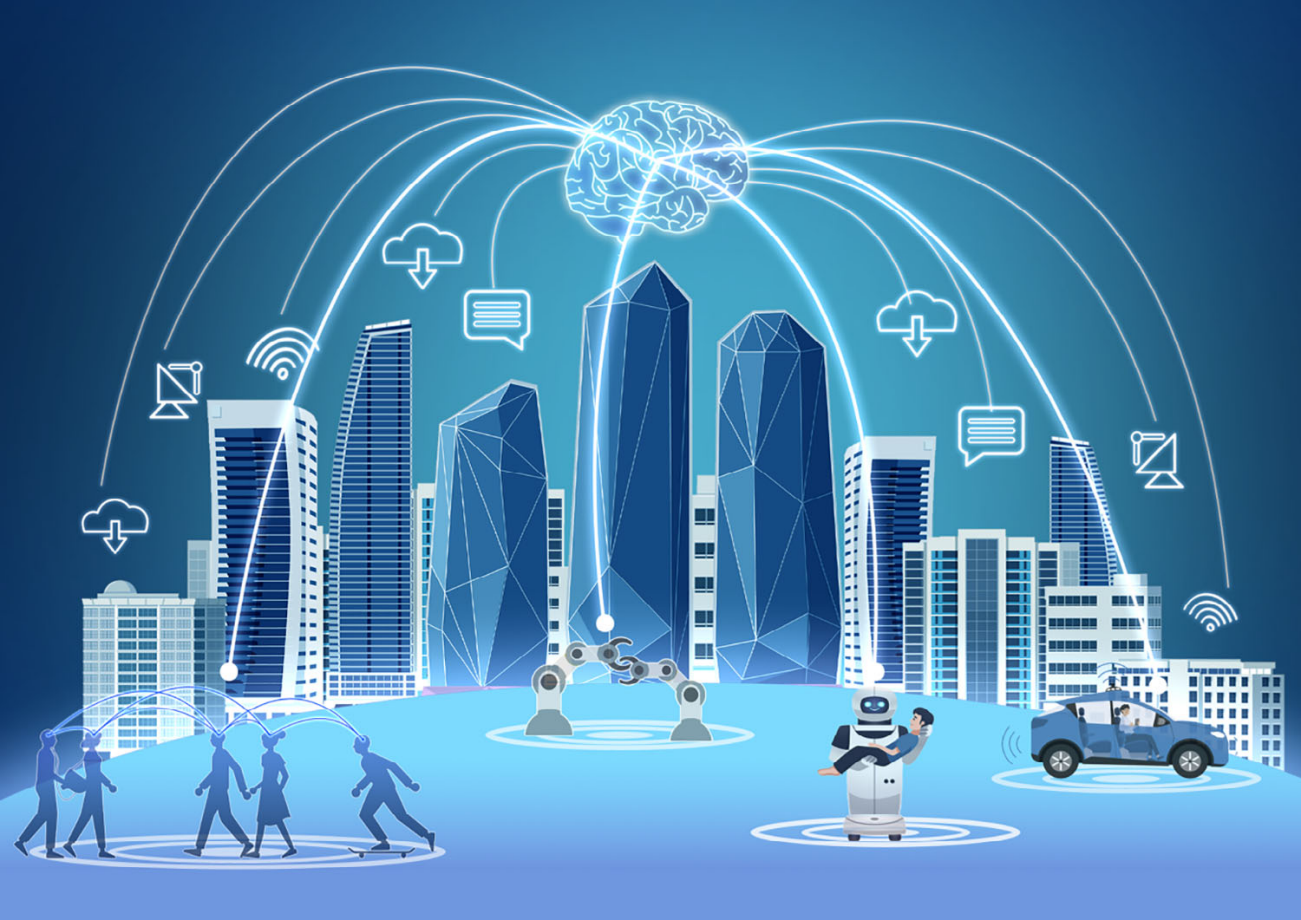
Perspective and possible designs on neural interfaces for future smart city applications including IoT and remote control; mind connectivity; limb and robot control; neural diseases treatments; traffic safety; and self‐driving vehicles.

Neural interfaces in the brain, including MEA, DBS, ECoG, and EEG are ushering in a revolutionary era that extends beyond healthcare into diverse realms of human‐technology interaction. These interfaces are pivotal in decoding brain signals, reshaping our communication with the external world, and integrating human cognition with urban technology. They offer a new level of autonomy, particularly for individuals with disabilities or mobility constraints (Figure 11). However, understanding their wide‐ranging implications for safety, inclusivity, and health is vital as they become integrated into future urban development.

Invasive neural interfaces (e.g. intracortical BMIs using action potentials and LFPs) offer direct and intuitive motor control, boasting higher accuracy and potential for fine motor command. However, they are subject to surgical risks and long‐term biocompatibility challenges, including nerve cell and blood vessel damage, infection, and immune rejection. Addressing these requires a deep understanding of the interaction between the tissue and foreign materials, and the development of biocompatible interfaces. Non‐invasive interfaces like EEG, while safer and less intrusive, offer lower performance due to their reliance on detecting broader neural activities and being subject to more noise and interference. Their future development hinges on improving signal processing and artifact removal techniques to enhance performance. The pursuit of high‐density electrodes aims to record from numerous neurons with high‐resolution signals, promising advancements in sensorimotor applications and beyond. However, these technologies still face challenges in miniaturization and signal crosstalk. A multidisciplinary approach involving neuroscience, engineering, psychology, and algorithm processing is essential to harness the full potential of these interfaces.

Initially, the primary application of neural interfaces was in rehabilitation and medical care to restore social interaction and movement capabilities in patients. The success of these applications has inspired the development of bidirectional and commercial BMIs. Technologies like cochlear implants represent successful examples of BMIs that have significantly enhanced human capabilities, although their interaction with the environment remains distinct from natural experiences. The future of BMIs lies in addressing the balance between precision and safety, with advancement in signal processing, miniaturization, and biocompatibility being critical. Intracortical BMIs promise higher information transfer rates and the potential for more natural control and feedback, including somatosensory feedback restoration. However, challenges in implant longevity, signal stability, and ethical considerations remain.

As BMIs advance, they raise significant ethical and societal questions regarding privacy, the potential for mind communication, and brain enhancement implications. The lack of specific standards for the development and use of these technologies poses risks of unauthorized access to sensitive brain signals. Rigorous standards for data acquisition, access control, and encryption should be established to protect user privacy. The path forward involves not only technological advancements but also a robust ethical framework, multidisciplinary collaboration, and regulatory oversight. Overcoming these challenges will enable neural interfaces to enhance human capabilities and quality of life responsibly and inclusively.

## CONFLICT OF INTEREST STATEMENT

The authors declare no conflicts of interest.

## Supporting information

Supporting Information

## References

[1] H. I. Chen , J. A. Wolf , R. Blue , M. M. Song , J. D. Moreno , G.‐l. Ming , H. Song , Cell Stem Cell 2019, 25, 462. 31585092 10.1016/j.stem.2019.09.002 PMC7180006

[2] I. Pediaditakis , K. R. Kodella , D. V. Manatakis , C. Y. Le , C. D. Hinojosa , W. Tien‐Street , E. S. Manolakos , K. Vekrellis , G. A. Hamilton , L. Ewart , Nat. Commun. 2021, 12, 5907. 34625559 10.1038/s41467-021-26066-5 PMC8501050

[3] J. Q. Yang , R. Wang , Y. Ren , J. Y. Mao , Z. P. Wang , Y. Zhou , S. T. Han , Adv. Mater. 2020, 32, 2003610. 10.1002/adma.202003610 33165986

[4] Y. Liu , W. Wang , D. Zhang , Y. Sun , F. Li , M. Zheng , D. B. Lovejoy , Y. Zou , B. Shi , Exploration 2022, 2, 20210274. 37325609 10.1002/EXP.20210274 PMC10190947

[5] J.‐S. Lim , H. J. Kim , I. Park , S. Woo , J.‐H. Kim , J. W. Park , Nano Lett. 2022, 22, 3865. 35549313 10.1021/acs.nanolett.1c04395

[6] A. G. Yearley , R. V. Patel , S. E. Blitz , S. Park , A. M. Madinger , J. Li , B. R. Johnston , P. P. Peruzzi , S. Lee , S. S. Srinivasan , Device 2023, 1, 100068.

[7] A. Zhang , Y. Jiang , K. Y. Loh , Z. Bao , K. Deisseroth , Nat. Rev. Bioeng. 2023, 2, 82.

[8] A. Greenberg , A. Cohen , M. Grewal , Nat. Biotechnol. 2021, 39, 1194. 34621062 10.1038/s41587-021-01071-7

[9] M. S. Willsey , S. R. Nason‐Tomaszewski , S. R. Ensel , H. Temmar , M. J. Mender , J. T. Costello , P. G. Patil , C. A. Chestek , Nat. Commun. 2022, 13, 6899. 36371498 10.1038/s41467-022-34452-w PMC9653378

[10] W. Heng , S. Solomon , W. Gao , Adv. Mater. 2022, 34, 2107902. 10.1002/adma.202107902 PMC9035141 34897836

[11] L. Drew , Nat. Electron. 2023, 6, 90.

[12] A. Nikitas , K. Michalakopoulou , E. T. Njoya , D. Karampatzakis , Sustainability 2020, 12, 2789.

[13] S. Lin , J. Liu , W. Li , D. Wang , Y. Huang , C. Jia , Z. Li , M. Murtaza , H. Wang , J. Song , Nano Lett. 2019, 19, 6853. 31454250 10.1021/acs.nanolett.9b02019

[14] X. Tang , H. Shen , S. Zhao , N. Li , J. Liu , Nat. Electron. 2023, 6, 109.

[15] R. Abiri , S. Borhani , E. W. Sellers , Y. Jiang , X. Zhao , J. Neural Eng. 2019, 16, 011001. 30523919 10.1088/1741-2552/aaf12e

[16] H. Acarón Ledesma , X. Li , J. L. Carvalho‐de‐Souza , W. Wei , F. Bezanilla , B. Tian , Nat. Nanotechnol. 2019, 14, 645. 31270446 10.1038/s41565-019-0487-x PMC6800006

[17] Q. Zeng , Z. Huang , Adv. Funct. Mater. 2023, 33, 2301223.

[18] Y. Duan , S. Wang , Q. Yuan , Y. Shi , N. Jiang , D. Jiang , J. Song , P. Wang , L. Zhuang , Small 2023, 19, 2205768. 10.1002/smll.202205768 37035943

[19] G. T. Go , Y. Lee , D. G. Seo , T. W. Lee , Adv. Mater. 2022, 34, 2201864. 10.1002/adma.202201864 35925610

[20] H. Song , M. Kim , E. Kim , J. Lee , I. Jeong , K. Lim , S. Y. Ryu , M. Oh , Y. Kim , J. U. Park , BMEMat 2023, DOI: 10.1002/bmm2.12048

[21] N. Wu , S. Wan , S. Su , H. Huang , G. Dou , L. Sun , InfoMat 2021, 3, 1174.

[22] K. Shen , O. Chen , J. L. Edmunds , D. K. Piech , M. M. Maharbiz , Nat. Biomed. Eng. 2023, 7, 424. 37081142 10.1038/s41551-023-01021-5

[23] S. Xu , M. Momin , S. Ahmed , A. Hossain , L. Veeramuthu , A. Pandiyan , C. C. Kuo , T. Zhou , Adv. Mater. 2023, 35, 2303267. 10.1002/adma.202303267 37726261

[24] M. Bianchi , A. De Salvo , M. Asplund , S. Carli , M. Di Lauro , A. Schulze‐Bonhage , T. Stieglitz , L. Fadiga , F. Biscarini , Adv. Sci. 2022, 9, 2104701. 10.1002/advs.202104701 PMC9036021 35191224

[25] J. Wang , T. Wang , H. Liu , K. Wang , K. Moses , Z. Feng , P. Li , W. Huang , Adv. Mater. 2023, 35, 2211012. 10.1002/adma.202211012 37143288

[26] R. A. Andersen , T. Aflalo , L. Bashford , D. Bjånes , S. Kellis , Annu. Rev. Psychol. 2022, 73, 131. 34982594 10.1146/annurev-psych-030221-030214

[27] Y. Cho , H. H. Jeong , H. Shin , C. J. Pak , J. Cho , Y. Kim , D. Kim , T. Kim , H. Kim , S. Kim , Adv. Sci. 2023, 10, 2303728. 10.1002/advs.202303728 PMC10724394 37840396

[28] B. B. Murphy , N. V. Apollo , P. Unegbu , T. Posey , N. Rodriguez‐Perez , Q. Hendricks , F. Cimino , A. G. Richardson , F. Vitale , iScience 2022, 25, 104652. 35811842 10.1016/j.isci.2022.104652 PMC9263525

[29] B. Hou , X. Liu , BMEMat 2023, 1, e12054.

[30] A. An , IET Smart Cities 2023, 5, 64.

[31] M. K. Algnbri , J. Metaverse 2022, 2, 29.

[32] G. Wang , A. Badal , X. Jia , J. S. Maltz , K. Mueller , K. J. Myers , C. Niu , M. Vannier , P. Yan , Z. Yu , Nat. Mach. Intell. 2022, 4, 922. 36935774 10.1038/s42256-022-00549-6 PMC10015955

[33] Y. Zhou , X. Xiao , G. Chen , X. Zhao , J. Chen , Joule 2022, 6, 1381.

[34] X. Zhao , H. Askari , J. Chen , Joule 2021, 5, 1391.

[35] A. Libanori , G. Chen , X. Zhao , Y. Zhou , J. Chen , Nat. Electron. 2022, 5, 142. 10.1038/s41928-022-00723-z PMC13309183 42367254

[36] G. Chen , X. Xiao , X. Zhao , T. Tat , M. Bick , J. Chen , Chem. Rev. 2021, 122, 3259. 34939791 10.1021/acs.chemrev.1c00502

[37] T. Venkatesan , S. Williams , Appl. Phys. Rev. 2022, 9, 010401.

[38] X. Zhang , L. Yao , S. Zhang , S. Kanhere , M. Sheng , Y. Liu , IEEE IoT J. 2018, 6, 2084.

[39] M. M. Shanechi , Nat. Neurosci. 2019, 22, 1554. 31551595 10.1038/s41593-019-0488-y

[40] P. Gagana , S. Kumar , S. Meghana , M. Advithi , S. Airaddi , J. Remote Sens. GIS Technol. 2022, 8, 10.

[41] F. Putze , D. Weiß , L.‐M. Vortmann , T. Schultz , in *IEEE Int. Conf. Syst. Man Cybern*., IEEE, Bari, Italy **2019**, 2812.

[42] J. P. Donoghue , Nat. Neurosci. 2002, 5, 1085. 12403992 10.1038/nn947

[43] W. Sun , J.‐W. Lin , S.‐F. Su , N. Wang , M. J. Er , IEEE Trans. Cybern. 2020, 51, 1099. 10.1109/TCYB.2020.2972582 32112693

[44] E. D. Oña , J. M. Garcia‐Haro , A. Jardón , C. Balaguer , Appl. Sci. 2019, 9, 2586.

[45] Y. Zhou , H. Song , G.‐l. Ming , Nat. Rev. Genet. 2024, 25, 26. 37507490 10.1038/s41576-023-00626-5 PMC10926850

[46] E. D'Angelo , V. Jirsa , Trends Neurosci. 2022, 45, 777. 35906100 10.1016/j.tins.2022.06.007

[47] N. C. Rust , J. E. LeDoux , Trends Neurosci. 2023, 46, 3. 36428194 10.1016/j.tins.2022.10.011

[48] Y.‐Y. Tang , R. Tang , M. I. Posner , J. J. Gross , Trends Cogn. Sci. 2022, 26, 567. 35537920 10.1016/j.tics.2022.04.006 PMC9625113

[49] W. Mildenberger , S. A. Stifter , M. Greter , Curr. Opin. Immunol. 2022, 76, 102181. 35462276 10.1016/j.coi.2022.102181

[50] M. Chini , I. L. Hanganu‐Opatz , Trends Neurosci. 2021, 44, 227. 33246578 10.1016/j.tins.2020.10.017

[51] J. L. Luby , T. Z. Baram , C. E. Rogers , D. M. Barch , Trends Neurosci. 2020, 43, 744. 32863044 10.1016/j.tins.2020.08.001 PMC7530018

[52] M. Banwinkler , H. Theis , S. Prange , T. van Eimeren , Brain Sci. 2022, 12, 1248. 36138882 10.3390/brainsci12091146 PMC9496752

[53] F. Svara , D. Förster , F. Kubo , M. Januszewski , M. Dal Maschio , P. J. Schubert , J. Kornfeld , A. A. Wanner , E. Laurell , W. Denk , Nat. Methods 2022, 19, 1357. 36280717 10.1038/s41592-022-01621-0 PMC9636024

[54] P. Gloor , Am. J. EEG Technol. 1969, 9, 1.

[55] J.‐P. Banquet , Electroencephalogr. Clin. Neurophysiol. 1973, 35, 143. 4124606 10.1016/0013-4694(73)90170-3

[56] W. N. Kuhlman , Electroencephalogr. Clin. Neurophysiol. 1978, 44, 83. 74329 10.1016/0013-4694(78)90107-4

[57] G. Pfurtscheller , A. Aranibar , Electroencephalogr. Clin. Neurophysiol. 1977, 42, 817. 67933 10.1016/0013-4694(77)90235-8

[58] A. Carmon , Y. Friedman , R. Coger , B. Kenton , Pain 1980, 8, 21. 7367035 10.1016/0304-3959(80)90087-1

[59] P. Hammond , A. Smith , J. Physiol. 1983, 342, 35. 6631738 10.1113/jphysiol.1983.sp014838 PMC1193946

[60] M. J. Hawken , A. Parker , J. Lund , J. Neurosci. 1988, 8, 3541. 3193169 10.1523/JNEUROSCI.08-10-03541.1988 PMC6569616

[61] A. Gevins , M. E. Smith , H. Leong , L. McEvoy , S. Whitfield , R. Du , G. Rush , Hum. Factors. 1998, 40, 79. 9579105 10.1518/001872098779480578

[62] A. Kostov , M. Polak , IEEE Trans. Rehabil. Eng. 2000, 8, 203. 10896187 10.1109/86.847816

[63] G. Pfurtscheller , C. Neuper , A. Schlogl , K. Lugger , IEEE Trans. Rehabil. Eng. 1998, 6, 316. 9749909 10.1109/86.712230

[64] S. Meystre , Telemed. J. E Health. 2005, 11, 63. 15785222 10.1089/tmj.2005.11.63

[65] T. Zaehle , S. Rach , C. S. Herrmann , PloS One 2010, 5, e13766. 21072168 10.1371/journal.pone.0013766 PMC2967471

[66] M. Tamietto , B. De Gelder , Nat. Rev. Neurosci. 2010, 11, 697. 20811475 10.1038/nrn2889

[67] U. Raghavendra , U. R. Acharya , H. Adeli , Eur. Neurol. 2020, 82, 41. 10.1159/000504292 31743905

[68] J. H. Shin , J. Kwon , J. U. Kim , H. Ryu , J. Ok , S. Joon Kwon , H. Park , T.‐I. Kim , npj Flex. Electron. 2022, 6, 32.

[69] J. Viventi , D.‐H. Kim , L. Vigeland , E. S. Frechette , J. A. Blanco , Y.‐S. Kim , A. E. Avrin , V. R. Tiruvadi , S.‐W. Hwang , A. C. Vanleer , Nat. Neurosci. 2011, 14, 1599. 22081157 10.1038/nn.2973 PMC3235709

[70] D. Khodagholy , J. N. Gelinas , T. Thesen , W. Doyle , O. Devinsky , G. G. Malliaras , G. Buzsáki , Nat. Neurosci. 2015, 18, 310. 25531570 10.1038/nn.3905 PMC4308485

[71] C. Harjes , K. Schoenbach , G. Schaefer , M. Kristiansen , H. Krompholz , D. Skaggs , Rev. Sci. Instrum. 1984, 55, 1684.

[72] E. M. Maynard , C. T. Nordhausen , R. A. Normann , Electroencephalogr. Clin. Neurophysiol. 1997, 102, 228. 9129578 10.1016/s0013-4694(96)95176-0

[73] T. J. Blanche , M. A. Spacek , J. F. Hetke , N. V. Swindale , J. Neurophysiol. 2005, 93, 2987. 15548620 10.1152/jn.01023.2004

[74] J. Lee , I. Ozden , Y.‐K. Song , A. V. Nurmikko , Nat. Methods 2015, 12, 1157. 26457862 10.1038/nmeth.3620

[75] E. A. Spiegel , H. T. Wycis , M. Marks , A. J. Lee , Science 1947, 106, 349. 17777432 10.1126/science.106.2754.349

[76] E. Bate‐Smith , Nature 1948, 161, 835. 18862127 10.1038/161835a0

[77] L. Leksell , Acta Chir Scand. 1949, 99, 229. 15391137

[78] D. E. Richardson , H. Akil , J. Neurosurg. 1977, 47, 184. 301558 10.3171/jns.1977.47.2.0184

[79] C. A. Gleason , B. L. Wise , B. Feinstein , Neurosurg. 1978, 2, 217. 10.1227/00006123-197805000-00006 215935

[80] G. Dieckmann , A. Witzmann , Stereotact. Funct. Neurosurg. 1982, 45, 167.

[81] A.‐L. Benabid , P. Pollak , A. Louveau , S. Henry , J. De Rougemont , Stereotact. Funct. Neurosurg. 1987, 50, 344. 10.1159/000100803 3329873

[82] J. Holsheimer , B. Nuttin , G. W. King , W. A. Wesselink , J. M. Gybels , P. De Sutter , Neurosurg. 1998, 42, 541. 10.1097/00006123-199803000-00022 9526989

[83] J. Volkmann , J. Herzog , F. Kopper , G. Deuschl , Mov. Disord. 2002, 17, S181. 11948775 10.1002/mds.10162

[84] D. A. Malone Jr , D. D. Dougherty , A. R. Rezai , L. L. Carpenter , G. M. Friehs , E. N. Eskandar , S. L. Rauch , S. A. Rasmussen , A. G. Machado , C. S. Kubu , Biol. Psychiatry 2009, 65, 267. 18842257 10.1016/j.biopsych.2008.08.029 PMC3486635

[85] S. Little , A. Pogosyan , S. Neal , B. Zavala , L. Zrinzo , M. Hariz , T. Foltynie , P. Limousin , K. Ashkan , J. FitzGerald , Ann. Neurol. 2013, 74, 449. 23852650 10.1002/ana.23951 PMC3886292

[86] S. Li , W. Zhou , Q. Yuan , Y. Liu , IEEE Trans. Neural Syst. Rehabil. Eng. 2013, 21, 880. 24122570 10.1109/TNSRE.2013.2282153

[87] D. N. Anderson , C. Anderson , N. Lanka , R. Sharma , C. R. Butson , B. W. Baker , A. D. Dorval , Front. Neurosci. 2019, 13, 1152. 31736693 10.3389/fnins.2019.01152 PMC6828644

[88] A. Z. Kouzani , O. A. Abulseoud , S. J. Tye , M. K. Hosain , M. Berk , IEEE J. Transl. Eng. Health Med. 2013, 1, 1500109. 10.1109/JTEHM.2013.2264093 PMC4819234 27170861

[89] F. Steigerwald , L. Müller , S. Johannes , C. Matthies , J. Volkmann , Mov. Disord. 2016, 31, 1240. 27241197 10.1002/mds.26669 PMC5089579

[90] H. Cagnan , T. Denison , C. McIntyre , P. Brown , Nat. Biotechnol. 2019, 37, 1024. 31477926 10.1038/s41587-019-0244-6 PMC6877347

[91] A. Boutet , I. Hancu , U. Saha , A. Crawley , D. S. Xu , M. Ranjan , E. Hlasny , R. Chen , W. Foltz , F. Sammartino , J. Neurosurg. 2019, 132, 586. 30797197 10.3171/2018.11.JNS181338

[92] S. R. Potel , S. Marceglia , S. Meoni , S. K. Kalia , R. G. Cury , E. Moro , Curr. Neurol. Neurosci. Rep. 2022, 22, 577. 35838898 10.1007/s11910-022-01221-7

[93] N. G. Pozzi , I. U. Isaias , Handb. Clin. Neurol. 2022, 184, 273. 35034741 10.1016/B978-0-12-819410-2.00015-1

[94] M. Wu , K. Yao , N. Huang , H. Li , J. Zhou , R. Shi , J. Li , X. Huang , J. Li , H. Jia , Adv. Sci. 2023, 10, 2300504. 10.1002/advs.202300504 PMC10190644 36825679

[95] X. Zhang , C. Wu , Y. Lv , Y. Zhang , W. Liu , Nano Lett. 2022, 22, 7246. 35984717 10.1021/acs.nanolett.2c02765

[96] Y. Huang , H. Li , T. Hu , J. Li , C. K. Yiu , J. Zhou , J. Li , X. Huang , K. Yao , X. Qiu , Nano Lett. 2022, 22, 5944. 35816764 10.1021/acs.nanolett.2c01997

[97] R. Tibon , L. Geerligs , K. Campbell , Trends Neurosci. 2022, 45, 507. 35469691 10.1016/j.tins.2022.03.011

[98] A. de Borman , S. Vespa , R. El Tahry , P.‐A. Absil , J. Neural Eng. 2022, 19, 026005. 10.1088/1741-2552/ac55ad 35172295

[99] W. Yan , Y. Wu , Front. Neurosci. 2022, 16, 991136. 36507356 10.3389/fnins.2022.991136 PMC9732370

[100] Q. Gong , Y. Yu , L. Kang , M. Zhang , Y. Zhang , S. Wang , Y. Niu , Y. Zhang , J. Di , Q. Li , Adv. Funct. Mater. 2022, 32, 2107360.

[101] J. Zhang , L. Wang , Y. Xue , I. M. Lei , X. Chen , P. Zhang , C. Cai , X. Liang , Y. Lu , J. Liu , Adv. Mater. 2023, 35, 2209324. 10.1002/adma.202209324 36398434

[102] P. Bourceau , B. Geier , V. Suerdieck , T. Bien , J. Soltwisch , K. Dreisewerd , M. Liebeke , Nat. Protoc. 2023, 18, 3050. 37674095 10.1038/s41596-023-00864-1

[103] I. K. Han , K. I. Song , S. M. Jung , Y. Jo , J. Kwon , T. Chung , S. Yoo , J. Jang , Y. T. Kim , D. S. Hwang , Adv. Mater. 2023, 35, 2203431. 10.1002/adma.202203431 35816086

[104] G. Tian , D. Yang , C. Liang , Y. Liu , J. Chen , Q. Zhao , S. Tang , J. Huang , P. Xu , Z. Liu , Adv. Mater. 2023, 35, 2212302. 10.1002/adma.202212302 36739173

[105] W. Yang , X. Kang , X. Gao , Y. Zhuang , C. Fan , H. Shen , Y. Chen , J. Dai , Adv. Funct. Mater. 2023, 33, 2211340.

[106] C. Yang , Z. Suo , Nat. Rev. Mater. 2018, 3, 125.

[107] Y. Fu , Y. Zhou , X. Huang , B. Dong , F. Zhuge , Y. Li , Y. He , Y. Chai , X. Miao , Adv. Funct. Mater. 2022, 32, 2111996.

[108] X. S. Zheng , Q. Yang , A. Vazquez , X. T. Cui , iScience 2022, 25, 104539. 35769881 10.1016/j.isci.2022.104539 PMC9234710

[109] C. Lee , B. Kim , J. Kim , S. Lee , T. Jeon , W. Choi , S. Yang , J.‐H. Ahn , J. Bae , Y. Chae , IEEE J. Solid‐State Circuits. 2022, 57, 3212.

[110] Y. Qiang , W. Gu , Z. Liu , S. Liang , J. H. Ryu , K. J. Seo , W. Liu , H. Fang , Nano Res. 2021, 14, 3240. 34394850 10.1007/s12274-021-3442-8 PMC8361849

[111] Y. Zhang , Q. Lu , J. He , Z. Huo , R. Zhou , X. Han , M. Jia , C. Pan , Z. L. Wang , J. Zhai , Nat. Commun. 2023, 14, 1252. 36878931 10.1038/s41467-023-36885-3 PMC9988987

[112] Y. Y. Chang , J. He , D. Pu , L. Chen , Y.‐M. Zhang , S. X.‐A. Zhang , Device 2023, 1, 100021.

[113] Z. Liu , L. Bai , Z. Zhu , L. Chen , Q. Sun , in *IEEE Electron. Compon. Technol. Conf*., IEEE Denver, United States **2022**, 2078.

[114] D. Jiang , A. Demosthenous , IEEE Trans. Biomed. Circuits Syst. 2018, 12, 940. 29993559 10.1109/TBCAS.2018.2832541

[115] R. Wilke , G. K. Moghadam , N. Lovell , G. Suaning , S. Dokos , J. Neural Eng. 2011, 8, 046016. 21673395 10.1088/1741-2560/8/4/046016

[116] I. B. Dimov , M. Moser , G. G. Malliaras , I. McCulloch , Chem. Rev. 2022, 122, 4356. 35089012 10.1021/acs.chemrev.1c00685 PMC9007464

[117] X. Gao , Y. Bao , Z. Chen , J. Lu , T. Su , L. Zhang , J. Ouyang , Adv. Electron. Mater. 2023, 9, 2300082.

[118] Q. Li , C. Wen , J. Yang , X. Zhou , Y. Zhu , J. Zheng , G. Cheng , J. Bai , T. Xu , J. Ji , Chem. Rev. 2022, 122, 17073. 36201481 10.1021/acs.chemrev.2c00344

[119] S. Kosuri , C. H. Borca , H. Mugnier , M. Tamasi , R. A. Patel , I. Perez , S. Kumar , Z. Finkel , R. Schloss , L. Cai , Adv. Healthc. Mater. 2022, 11, 2102101. 10.1002/adhm.202102101 PMC9119153 35112508

[120] M. Stephen , A. Nawaz , S. Y. Lee , P. Sonar , W. L. Leong , Adv. Funct. Mater. 2023, 33, 2208521.

[121] J. Snider , M. Plank , G. Lynch , E. Halgren , H. Poizner , J. Neurosci. 2013, 33, 15056. 24048836 10.1523/JNEUROSCI.0268-13.2013 PMC3776058

[122] J. O'Byrne , K. Jerbi , Trends Neurosci. 2022, 45, 820. 36096888 10.1016/j.tins.2022.08.007

[123] K. L. Ray , N. R. Griffin , J. Shumake , A. Alario , J. J. Allen , C. G. Beevers , D. M. Schnyer , Brain Res. 2023, 1806, 148282. 36792002 10.1016/j.brainres.2023.148282

[124] T. Onojima , T. Goto , H. Mizuhara , T. Aoyagi , PLoS Comput. Biol. 2018, 14, e1005928. 29337999 10.1371/journal.pcbi.1005928 PMC5770039

[125] C. B. Holroyd , Trends Neurosci. 2022, 45, 346. 35236639 10.1016/j.tins.2022.02.002

[126] R. Coa , S. M. La Cava , G. Baldazzi , L. Polizzi , G. Pinna , C. Conti , G. Defazio , D. Pani , M. Puligheddu , Front. Neurol. 2022, 13, 1030118. 36504670 10.3389/fneur.2022.1030118 PMC9728998

[127] X. Yang , Z. Chen , Z. Wang , G. He , Z. Li , Y. Shi , N. Gong , B. Zhao , Y. Kuang , E. Takahashi , Mol. Brain 2022, 15, 16. 35144651 10.1186/s13041-022-00901-2 PMC8832845

[128] J. Royer , B. C. Bernhardt , S. Larivière , E. Gleichgerrcht , B. J. Vorderwülbecke , S. Vulliémoz , L. Bonilha , Epilepsia 2022, 63, 537. 35092011 10.1111/epi.17171

[129] D. Yang , Q. Ren , J. Nie , Y. Zhang , H. Wu , Z. Chang , B. Wang , J. Dai , Y. Fang , Nano Lett. 2023, 24, 1052. 37955335 10.1021/acs.nanolett.3c03472

[130] C. Guger , S. Daban , E. Sellers , C. Holzner , G. Krausz , R. Carabalona , F. Gramatica , G. Edlinger , Neurosci. Lett. 2009, 462, 94. 19545601 10.1016/j.neulet.2009.06.045

[131] A. Criscuolo , M. Schwartze , S. A. Kotz , Trends Neurosci. 2022, 45, 667. 35810022 10.1016/j.tins.2022.06.004

[132] C. Adaikkan , L.‐H. Tsai , Trends Neurosci. 2020, 43, 24. 31836315 10.1016/j.tins.2019.11.001

[133] A. Benussi , V. Cantoni , M. Grassi , L. Brechet , C. M. Michel , A. Datta , C. Thomas , S. Gazzina , M. S. Cotelli , M. Bianchi , Ann. Neurol. 2022, 92, 322. 35607946 10.1002/ana.26411 PMC9546168

[134] R. Zheng , Z. Wang , Y. He , J. Zhang , Cogn. Neurodyn. 2022, 16, 325. 35401867 10.1007/s11571-021-09714-w PMC8934897

[135] P. Li , C. Wang , M. Li , X. Xuan , B. Zhou , H. Li , Adv. Intell. Syst. 2023, 5, 2300018.

[136] B. Voytek , Nat. Methods 2022, 19, 1349. 36203017 10.1038/s41592-022-01630-z PMC9637751

[137] D. Borra , S. Fantozzi , M. C. Bisi , E. Magosso , Sensors 2023, 23, 3530. 37050590 10.3390/s23073530 PMC10099070

[138] E. E. Asher , M. Plotnik , M. Günther , S. Moshel , O. Levy , S. Havlin , J. W. Kantelhardt , R. P. Bartsch , Commun. Biol. 2021, 4, 1017. 34462540 10.1038/s42003-021-02544-w PMC8405655

[139] C. Strauch , C.‐A. Wang , W. Einhäuser , S. Van der Stigchel , M. Naber , Trends Neurosci. 2022, 45, 635. 35662511 10.1016/j.tins.2022.05.003

[140] K. J. Clancy , S. K. Baisley , A. Albizu , N. Kartvelishvili , M. Ding , W. Li , Soc. Cogn. Affect. Neurosci. 2018, 13, 1305. 30380131 10.1093/scan/nsy096 PMC6277743

[141] R. Kaveh , J. Doong , A. Zhou , C. Schwendeman , K. Gopalan , F. L. Burghardt , A. C. Arias , M. M. Maharbiz , R. Muller , IEEE Trans. Biomed. Circuits Syst. 2020, 14, 727. 32746342 10.1109/TBCAS.2020.3001265

[142] J. J. Norton , D. S. Lee , J. W. Lee , W. Lee , O. Kwon , P. Won , S.‐Y. Jung , H. Cheng , J.‐W. Jeong , A. Akce , Proc. Natl. Acad. Sci. U. S. A. 2015, 112, 3920. 25775550 10.1073/pnas.1424875112 PMC4386388

[143] K. E. Bouchard , N. Mesgarani , K. Johnson , E. F. Chang , Nature 2013, 495, 327. 23426266 10.1038/nature11911 PMC3606666

[144] G. Schalk , E. C. Leuthardt , IEEE Rev. Biomed. Eng. 2011, 4, 140. 22273796 10.1109/RBME.2011.2172408

[145] Y. Yu , Y. Qiu , G. Li , K. Zhang , B. Bo , M. Pei , J. Ye , G. J. Thompson , J. Cang , F. Fang , Y. Feng , X. Duan , C. Tong , Z. Liang , Nat. Commun. 2023, 14, 1651. 36964161 10.1038/s41467-023-37352-9 PMC10039056

[146] L. L. Tan , M. J. Oswald , R. Kuner , Trends Neurosci. 2021, 44, 629. 34176645 10.1016/j.tins.2021.05.003

[147] A. Imai , S. Takahashi , S. Furubayashi , Y. Mizuno , M. Sonoda , T. Miyazaki , E. Miyashita , T. Fujie , Adv. Mater. Technol. 2023, 8, 2300300.

[148] E. C. Leuthardt , G. Schalk , J. R. Wolpaw , J. G. Ojemann , D. W. Moran , J. Neural Eng. 2004, 1, 63. 15876624 10.1088/1741-2560/1/2/001

[149] P. Asman , S. Prabhu , S. Tummala , N. F. Ince , in *Proc. 44th Annu. Int. Conf. IEEE Eng. Med. Biol. Soc*., IEEE, Glasgow, United Kingdom 2022, 4892. 10.1109/EMBC48229.2022.9871900 36085684

[150] M. O. Owolabi , M. Leonardi , C. Bassetti , J. Jaarsma , T. Hawrot , A. I. Makanjuola , R. K. Dhamija , W. Feng , V. Straub , J. Camaradou , Nat. Rev. Neurol. 2023, 19, 371. 37208496 10.1038/s41582-023-00808-z PMC10197060

[151] R. F. Betzel , J. D. Medaglia , A. E. Kahn , J. Soffer , D. R. Schonhaut , D. S. Bassett , Nat. Biomed. Eng. 2019, 3, 902. 31133741 10.1038/s41551-019-0404-5

[152] E. S. JeyaJothi , J. Anitha , S. Rani , B. Tiwari , Biomed. Res. Int. 2022, 2022, 7242667. 35224099 10.1155/2022/7242667 PMC8866013

[153] Z. Ye , C. Hu , J. Wang , H. Liu , L. Li , J. Yuan , J. W. Ha , Z. Li , L. Xiao , Exploration 2023, 3, 2023002. 10.1002/EXP.20230002 PMC10582609 37933279

[154] P. Zhang , W. Li , C. Liu , F. Qin , Y. Lu , M. Qin , Y. Hou , Exploration 2023, 3, 20230070. 38264683 10.1002/EXP.20230070 PMC10742208

[155] G. Hong , C. M. Lieber , Nat. Rev. Neurosci. 2019, 20, 330. 30833706 10.1038/s41583-019-0140-6 PMC6531316

[156] S. Liu , L. Liu , Y. Zhao , Y. Wang , Y. Wu , X.‐D. Zhang , D. Ming , Nano Lett. 2022, 22, 4400. 35587781 10.1021/acs.nanolett.2c00848

[157] N. V. Thakor , Sci. Tranl. Med. 2013, 5, 210. 10.1126/scitranslmed.3007303 24197734

[158] N. Ren , C. Hang , X. Liu , X. Jiang , Nano Lett. 2022, 22, 7554. 36122317 10.1021/acs.nanolett.2c02548

[159] L. Ferschmann , M. G. Bos , M. M. Herting , K. L. Mills , C. K. Tamnes , Curr. Opin. Psychol. 2022, 44, 170. 34688028 10.1016/j.copsyc.2021.09.014

[160] E. A. Mankin , I. Fried , Neuron 2020, 106, 218. 32325058 10.1016/j.neuron.2020.02.024 PMC7347298

[161] E. Lowet , K. Kondabolu , S. Zhou , R. A. Mount , Y. Wang , C. R. Ravasio , X. Han , Nat. Commun. 2022, 13, 7709. 36513664 10.1038/s41467-022-35314-1 PMC9748039

[162] W.‐J. Neumann , A. Horn , A. A. Kühn , Trends Neurosci. 2023, 46, 472. 37105806 10.1016/j.tins.2023.03.009

[163] F. Sun , H. Shen , Q. Yang , Z. Yuan , Y. Chen , W. Guo , Y. Wang , L. Yang , Z. Bai , Q. Liu , Adv. Mater. 2023, 35, 2210018. 10.1002/adma.202210018 36864009

[164] V. Emiliani , E. Entcheva , R. Hedrich , P. Hegemann , K. R. Konrad , C. Lüscher , M. Mahn , Z.‐H. Pan , R. R. Sims , J. Vierock , Nat. Rev. Methods Primers 2022, 2, 55. 37933248 10.1038/s43586-022-00136-4 PMC10627578

[165] M. A. Rossi , Trends Neurosci. 2023, 46, 738. 37353461 10.1016/j.tins.2023.05.010 PMC10524917

[166] R. Chen , G. Romero , M. G. Christiansen , A. Mohr , P. Anikeeva , Science 2015, 347, 1477. 25765068 10.1126/science.1261821

[167] A. M. Lozano , N. Lipsman , H. Bergman , P. Brown , S. Chabardes , J. W. Chang , K. Matthews , C. C. McIntyre , T. E. Schlaepfer , M. Schulder , Nat. Rev. Neurol. 2019, 15, 148. 30683913 10.1038/s41582-018-0128-2 PMC6397644

[168] B. Xiao , E.‐K. Tan , Trends Neurosci. 2023, 46, 1. 36207171 10.1016/j.tins.2022.09.004

[169] K. Udupa , R. Chen , Prog. Neurobiol. 2015, 133, 27. 26296674 10.1016/j.pneurobio.2015.08.001

[170] K. W. Scangos , A. N. Khambhati , P. M. Daly , G. S. Makhoul , L. P. Sugrue , H. Zamanian , T. X. Liu , V. R. Rao , K. K. Sellers , H. E. Dawes , Nat. Med. 2021, 27, 1696. 34608328 10.1038/s41591-021-01480-w PMC11219029

[171] H. Zhou , K.‐N. Kim , M.‐J. Sung , S. J. Han , T.‐W. Lee , Device 2023, 1, 100060.

[172] R. Ghanim , A. Kaushik , J. Park , A. Abramson , Device 2023, 1, 100092.

[173] L. Aron , J. Zullo , B. A. Yankner , Curr. Opin. Neurobiol. 2022, 72, 91. 34689041 10.1016/j.conb.2021.09.009 PMC8901453

[174] M. L. Kringelbach , N. Jenkinson , S. L. Owen , T. Z. Aziz , Nat. Rev. Neurosci. 2007, 8, 623. 17637800 10.1038/nrn2196

[175] M. Vissani , I. U. Isaias , A. Mazzoni , J. Neural Eng. 2020, 17, 051002. 33052884 10.1088/1741-2552/abb581

[176] J. Frey , J. Cagle , K. A. Johnson , J. K. Wong , J. D. Hilliard , C. R. Butson , M. S. Okun , C. de Hemptinne , Front. Neurol. 2022, 13, 825178. 35356461 10.3389/fneur.2022.825178 PMC8959612

[177] M. S. Messina , C. J. Chang , ACS Cent. Sci. 2023, 9, 1706. 37780366 10.1021/acscentsci.3c01070 PMC10540294

[178] J. A. Lecoq , R. Boehringer , B. F. Grewe , Nat. Methods 2023, 20, 495. 36869123 10.1038/s41592-023-01808-z

[179] A. Horn , M. Reich , J. Vorwerk , N. Li , G. Wenzel , Q. Fang , T. Schmitz‐Hübsch , R. Nickl , A. Kupsch , J. Volkmann , Ann. Neurol. 2017, 82, 67. 28586141 10.1002/ana.24974 PMC5880678

[180] J. K. Krauss , N. Lipsman , T. Aziz , A. Boutet , P. Brown , J. W. Chang , B. Davidson , W. M. Grill , M. I. Hariz , A. Horn , Nat. Rev. Neurol. 2021, 17, 75. 33244188 10.1038/s41582-020-00426-z PMC7116699

[181] T. Yanagisawa , R. Fukuma , B. Seymour , K. Hosomi , H. Kishima , T. Shimizu , H. Yokoi , M. Hirata , T. Yoshimine , Y. Kamitani , Nat. Commun. 2016, 7, 13209. 27807349 10.1038/ncomms13209 PMC5095287

[182] X. Liu , Y. Zhao , J. Dou , Q. Hou , J. Cheng , X. Jiang , Nano Lett. 2022, 22, 1091. 35089039 10.1021/acs.nanolett.1c04184

[183] S. Kikkert , J. Kolasinski , S. Jbabdi , I. Tracey , C. F. Beckmann , H. Johansen‐Berg , T. R. Makin , Elife 2016, 5, e15292. 27552053 10.7554/eLife.15292 PMC5040556

[184] L. Shan , H. Zeng , Y. Liu , X. Zhang , E. Li , R. Yu , Y. Hu , T. Guo , H. Chen , Nano Lett. 2022, 22, 7275. 36000976 10.1021/acs.nanolett.2c02995

[185] R. Godwin‐Jones , Lang. Learn. Technol. 2023, 27, 6.

[186] Q. Yang , R. Mishra , Y. Cen , G. Shi , R. Sharma , X. Fong , H. Yang , Nano Lett. 2022, 22, 8437. 36260522 10.1021/acs.nanolett.2c02409

[187] S. P. Padhy , S. V. Kalinin , Device 2023, 1, 100115.

[188] S. Schreiber , J. Bernal , P. Arndt , F. Schreiber , P. Müller , L. Morton , R. C. Braun‐Dullaeus , M. D. C. Valdés‐Hernández , R. Duarte , J. M. Wardlaw , Cell 2023, 12, 957. 10.3390/cells12060957 PMC10047140 36980297

[189] K. K. Ang , Z. Y. Chin , H. Zhang , C. Guan , in Proc. 2008 Int. Joint Conf. Neural Networks , IEEE, Hong Kong, China 2008, 2390.

[190] Y. Zhang , G. Zhou , J. Jin , X. Wang , A. Cichocki , J. Neurosci. Methods 2015, 255, 85. 26277421 10.1016/j.jneumeth.2015.08.004

[191] Z. Shuaibu , L. Qi , Int. J. Comput. Applic. 2020, 175, 16.

[192] S. Makeig , J. Onton , ERP Features and EEG Dynamics: An ICA Perspective. Oxford University Press, Oxford Handbooks Online 2011.

[193] X. Wu , B. Zhou , Z. Lv , C. Zhang , IEEE J. Biomed. Health Inf. 2019, 24, 775. 10.1109/JBHI.2019.2922976 31217132

[194] C.‐T. Lin , Y. Tian , Y.‐K. Wang , T.‐T. N. Do , Y.‐L. Chang , J.‐T. King , K.‐C. Huang , L.‐D. Liao , IEEE Trans. Intell. Transp. Syst. 2022, 23, 10395.

[195] V. Chamola , A. Vineet , A. Nayyar , E. Hossain , Sensors 2020, 20, 3620. 32605077 10.3390/s20133620 PMC7374399

[196] R. Atangana , D. Tchiotsop , G. Kenne , L. Chanel , Health Inf. Int. J. 2020, 9, 14.

[197] T. Siddharth , P. Gajbhiye , R. K. Tripathy , R. B. Pachori , IEEE Sens. J. 2020, 20, 11421.

[198] M. Sha'Abani , N. Fuad , N. Jamal , M. Ismail , in *Proc. 5th Int. Conf. Electr. Control Comput. Eng*., IEEE, Kuantan, Malaysia 2020, 555.

[199] M. Huang , Z. Liu , Y. Tao , Simul. Model. Pract. Theory 2020, 102, 101981.

[200] P.‐J. Lin , T. Jia , C. Li , T. Li , C. Qian , Z. Li , Y. Pan , L. Ji , IEEE Trans. Neural Syst. Rehabil. Eng. 2021, 29, 1936. 34516378 10.1109/TNSRE.2021.3112167

[201] T. Hosman , M. Vilela , D. Milstein , J. N. Kelemen , D. M. Brandman , L. R. Hochberg , J. D. Simeral , in *Proc. 9th Int. IEEE/EMBS Conf. Neural Eng*., IEEE, San Francisco, United States 2019, 1066.

[202] J. Guo , Y. Liu , L. Lin , S. Li , J. Cai , J. Chen , W. Huang , Y. Lin , J. Xu , Nano Lett. 2023, 23, 9651. 37548947 10.1021/acs.nanolett.3c02194

[203] K. K. Chan , L.‐W. Shang , Z. Qiao , Y. Liao , M. Kim , Y.‐C. Chen , Nano Lett. 2022, 22, 8949. 36367840 10.1021/acs.nanolett.2c03148

[204] J. G. Makin , D. A. Moses , E. F. Chang , Nat. Neurosci. 2020, 23, 575. 32231340 10.1038/s41593-020-0608-8 PMC10560395

[205] G. K. Anumanchipalli , J. Chartier , E. F. Chang , Nature 2019, 568, 493. 31019317 10.1038/s41586-019-1119-1 PMC9714519

[206] J. R. Wolpaw , N. Birbaumer , D. J. McFarland , G. Pfurtscheller , T. M. Vaughan , Clin. Neurophysiol. 2002, 113, 767. 12048038 10.1016/s1388-2457(02)00057-3

[207] L. A. Farwell , E. Donchin , Electroencephalogr. Clin. Neurophysiol. 1988, 70, 510. 2461285 10.1016/0013-4694(88)90149-6

[208] S. Luo , M. Angrick , C. Coogan , D. N. Candrea , K. Wyse‐Sookoo , S. Shah , Q. Rabbani , G. W. Milsap , A. R. Weiss , W. S. Anderson , Adv. Sci. 2023, 10, 202304853. 10.1002/advs.202304853 PMC10724434 37875404

[209] M. A. Schwemmer , N. D. Skomrock , P. B. Sederberg , J. E. Ting , G. Sharma , M. A. Bockbrader , D. A. Friedenberg , Nat. Med. 2018, 24, 1669. 30250141 10.1038/s41591-018-0171-y

[210] F. R. Willett , E. M. Kunz , C. Fan , D. T. Avansino , G. H. Wilson , E. Y. Choi , F. Kamdar , L. R. Hochberg , S. Druckmann , K. V. Shenoy , Nature 2023, 620, 1031. 37612500 10.1038/s41586-023-06377-x PMC10468393

[211] A. B. Rapeaux , T. G. Constandinou , Curr. Opin. Biotechnol. 2021, 72, 102. 34749248 10.1016/j.copbio.2021.10.001

[212] O. G. Sani , Y. Yang , M. M. Shanechi , BCI Res. 2021, 9, 121.

[213] Y. Yang , S. Qiao , O. G. Sani , J. I. Sedillo , B. Ferrentino , B. Pesaran , M. M. Shanechi , Nat. Biomed. Eng. 2021, 5, 324. 33526909 10.1038/s41551-020-00666-w

[214] F. Xia , M. A. Kheirbek , Trends Neurosci. 2020, 43, 902. 32917408 10.1016/j.tins.2020.08.004 PMC7606349

[215] J. Lee , H. Liao , Q. Wang , J. Han , J. H. Han , H. E. Shin , M. Ge , W. Park , F. Li , Exploration 2022, 2, 20210086. 37324577 10.1002/EXP.20210086 PMC10191057

[216] J. Mei , E. Muller , S. Ramaswamy , Trends Neurosci. 2022, 45, 237. 35074219 10.1016/j.tins.2021.12.008

[217] X. Wan , Z. Li , W. Yu , A. Wang , X. Ke , H. Guo , J. Su , L. Li , Q. Gui , S. Zhao , Adv. Mater. 2023, DOI: 10.1002/adma.202305192 37688451

[218] E. Kinney‐Lang , B. Auyeung , J. Escudero , J. Neural Eng. 2016, 13, 061002. 27762234 10.1088/1741-2560/13/6/061002

[219] T. Wang , Z. Song , X. Zhao , Y. Wu , L. Wu , A. Haghparast , H. Wu , Exploration 2023, 3, 20220133. 38264685 10.1002/EXP.20220133 PMC10742195

[220] Z. Wang , X. Yang , B. Zhao , W. Li , Heliyon 2023, 9, e14786. 37077680 10.1016/j.heliyon.2023.e14786 PMC10106918

[221] S. Fulton , L. Décarie‐Spain , X. Fioramonti , B. Guiard , S. Nakajima , Trends Endocrinol. Metab. 2022, 33, 18. 34750064 10.1016/j.tem.2021.10.005

[222] Z. Yuan , Y. Peng , L. Wang , S. Song , S. Chen , L. Yang , H. Liu , H. Wang , G. Shi , C. Han , IEEE Trans. Neural Syst. Rehabil. Eng. 2021, 29, 2569. 34871175 10.1109/TNSRE.2021.3132944

[223] O. G. Sani , Y. Yang , M. B. Lee , H. E. Dawes , E. F. Chang , M. M. Shanechi , Nat. Biotechnol. 2018, 36, 954. 30199076 10.1038/nbt.4200

[224] X. Wu , Y. Jiang , N. J. Rommelfanger , F. Yang , Q. Zhou , R. Yin , J. Liu , S. Cai , W. Ren , A. Shin , Nat. Biomed. Eng. 2022, 6, 754. 35314800 10.1038/s41551-022-00862-w PMC9232843

[225] H. Cui , S. Zhao , G. Hong , Device 2023, 1, 100113. 37990694 10.1016/j.device.2023.100113 PMC10659575

[226] M. Brennan , Device 2023, 1, 100096.

[227] A. Pandiyan , L. Veeramuthu , Z.‐L. Yan , Y.‐C. Lin , C.‐H. Tsai , S.‐T. Chang , W.‐H. Chiang , S. Xu , T. Zhou , C.‐C. Kuo , Prog. Mater. Sci. 2023, 140, 101206.

[228] Z. Dai , M. Lei , S. Ding , Q. Zhou , B. Ji , M. Wang , B. Zhou , Exploration 2023, 3, 20230046. 10.1002/EXP.20230046 PMC11022629 38855620

[229] T. Tat , X. Zhao , J. Chen , Device 2023, 1, 100094.

[230] L.‐D. Liao , C.‐Y. Chen , I.‐J. Wang , S.‐F. Chen , S.‐Y. Li , B.‐W. Chen , J.‐Y. Chang , C.‐T. Lin , J. Neuroeng. Rehabil. 2012, 9, 5. 22284235 10.1186/1743-0003-9-5 PMC3283495

[231] L. Piccini , S. Parini , L. Maggi , G. Andreoni , in *27th Annu. Int. Conf. IEEE Eng. Med. Biol. Soc*., IEEE, Piscataway, United States 2006, 5384.

[232] T. J. Sullivan , S. R. Deiss , T.‐P. Jung , G. Cauwenberghs , A brain‐machine interface using dry‐contact, low‐noise EEG sensors, in Proc. IEEE Int. Symp. Circuits Syst , IEEE Seattle, United States 2008, 1986.

[233] Y. Design , https://www.yankodesign.com/2021/02/23/cognixions (accessed February 2021).

[234] M. Jakobs , A. Fomenko , A. M. Lozano , K. L. Kiening , EMBO Mol. Med. 2019, 11, e9575. 30862663 10.15252/emmm.201809575 PMC6460356

[235] C. S. Mestais , G. Charvet , F. Sauter‐Starace , M. Foerster , D. Ratel , A. L. Benabid , IEEE Trans. Neural Syst. Rehabil. Eng. 2014, 23, 10. 25014960 10.1109/TNSRE.2014.2333541

[236] Fonds Clinatec , https://fonds‐clinatec.fr/projets‐projets‐soutenus/ (accessed June 2022).

[237] Z. Sun , M. Zhu , X. Shan , C. Lee , Nat. Commun. 2022, 13, 5224. 36064838 10.1038/s41467-022-32745-8 PMC9445040

[238] E. Leroy , R. Hinchet , H. Shea , Adv. Mater. 2020, 32, 2002564. 10.1002/adma.202002564 32700326

[239] M. Zhu , Z. Sun , Z. Zhang , Q. Shi , T. He , H. Liu , T. Chen , C. Lee , Sci. Adv. 2020, 6, eaaz8693. 32494718 10.1126/sciadv.aaz8693 PMC7209995

[240] Y. R. Lee , T. Q. Trung , B.‐U. Hwang , N.‐E. Lee , Nat. Commun. 2020, 11, 2753. 32488078 10.1038/s41467-020-16606-w PMC7265430

[241] J. Park , D.‐h. Kang , H. Chae , S. K. Ghosh , C. Jeong , Y. Park , S. Cho , Y. Lee , J. Kim , Y. Ko , Sci. Adv. 2022, 8, eabj9220. 35333568 10.1126/sciadv.abj9220 PMC8956263

[242] A. Halbach , P. Gijsenbergh , Y. Jeong , W. Devriese , H. Gao , M. Billen , G. B. Torri , C. Chare , D. Cheyns , X. Rottenberg , V. Rochus , In 20th Int. Conf. Solid‐State Sens. Actuat. Microsyst. Eurosens. XXXIII . IEEE, Estrel Berlin, Germany 2019, 158.

[243] T. H. Yang , J. R. Kim , H. Jin , H. Gil , J. H. Koo , H. J. Kim , Adv. Funct. Mater. 2021, 31, 2008831.

[244] Ultraleap, https://www.ultraleap.com/ (accessed June 2021).

[245] R. Hirayama , D.M. Plasencia , N. Masuda , S. Subramanian , Nature 2019, 575, 320. 31723288 10.1038/s41586-019-1739-5

[246] H. Yin , W. Jiang , Y. Liu , D. Zhang , F. Wu , Y. Zhang , C. Li , G. Chen , Q. Wang , BMEMat 2023, 1, e12023.

[247] H. Lee , J.‐S. Kim , S. Choi , J.‐H. Jun , J.‐R. Park , A.‐H. Kim , H.‐B. Oh , H.‐S. Kim , S.‐C. Chung , in *Proc. IEEE World Haptics Conf*. Evanston, IEEE, United States 2015, 374.

[248] Q. Zhang , H. Dong , A. El Saddik , IEEE Access 2016, 4, 299.

[249] K. Song , S. H. Kim , S. Jin , S. Kim , S. Lee , J.‐S. Kim , J.‐M. Park , Y. Cha , Sci. Rep. 2019, 9, 8988. 31320674 10.1038/s41598-019-45422-6 PMC6639318

[250] R. De Fazio , V. M. Mastronardi , M. Petruzzi , M. De Vittorio , P. Visconti , Future Internet 2022, 15, 14.

[251] M. Ying , A. P. Bonifas , N. Lu , Y. Su , R. Li , H. Cheng , A. Ameen , Y. Huang , J. A. Rogers , Nanotechnology 2012, 23, 344004. 22885907 10.1088/0957-4484/23/34/344004

[252] J. Lee , H. Sul , W. Lee , K. R. Pyun , I. Ha , D. Kim , H. Park , H. Eom , Y. Yoon , J. Jung , Adv. Funct. Mater. 2020, 30, 1909171.

[253] S. Choi , J. Park , W. Hyun , J. Kim , J. Kim , Y. B. Lee , C. Song , H. J. Hwang , J. H. Kim , T. Hyeon , ACS Nano 2015, 9, 6626. 26027637 10.1021/acsnano.5b02790

[254] S. Kim , T. Kim , C. S. Kim , H. Choi , Y. J. Kim , G. S. Lee , O. Oh , B. J. Cho , Soft Rob. 2020, 7, 736. 10.1089/soro.2019.0158 32286158

[255] S. Xu , S. Ahmed , M. Momin , A. Hossain , T. Zhou , Device 2023, 1, 100067.

[256] S. Xu , Z. Han , K. Yuan , P. Qin , W. Zhao , T. Lin , T. Zhou , F. Huang , Nat. Rev. Methods Primers 2023, 3, 44.

[257] H. Wang , S. Xu , W. Dong , D. Sun , S. Zhang , Z. Han , F. Huang , Chem. Eur. J. 2022, 28, e202200124. 35170808 10.1002/chem.202200124

[258] M. Beyeler , M. Sanchez‐Garcia , J. Neural Eng. 2022, 19, 063001. 10.1088/1741-2552/aca69d PMC10507809 36541463

[259] W. Zgallai , J. T. Brown , A. Ibrahim , F. Mahmood , K. Mohammad , M. Khalfan , M. Mohammed , M. Salem , N. Hamood , in Proc. Adv. Sci. Eng. Technol. Int. Conf., IEEE, Dubai, United Arab Emirates 2019, 1.

[260] A. M. Aslam , R. Chaudhary , A. Bhardwaj , I. Budhiraja , N. Kumar , S. Zeadally , IEEE IoT J. 2023, 6, 32.

[261] U. Chaudhary , N. Birbaumer , A. Ramos‐Murguialday , Nat. Rev. Neurol. 2016, 12, 513. 27539560 10.1038/nrneurol.2016.113

[262] A. Chella , E. Pagello , E. Menegatti , R. Sorbello , S. M. Anzalone , F. Cinquegrani , L. Tonin , F. Piccione , K. Prifitis , C. Blanda , in *Proc. Int. Conf. Complex Int. Softw. Intensive Syst*., IEEE, Fukuoka, Japan 2009, 783.

[263] R. Spataro , A. Chella , B. Allison , M. Giardina , R. Sorbello , S. Tramonte , C. Guger , V. La Bella , Front. Hum. Neurosci. 2017, 11, 68. 28298888 10.3389/fnhum.2017.00068 PMC5331030

[264] S.‐M. Xu , X. Liang , X.‐Y. Wu , S.‐L. Zhao , J. Chen , K.‐X. Wang , J.‐S. Chen , Nat. Commun. 2019, 10, 5810. 31862935 10.1038/s41467-019-13712-2 PMC6925149

[265] S. Xu , B. Peng , X. Pang , F. Huang , ACS Mater. Lett. 2022, 4, 2195.

[266] S.‐M. Xu , X. Liang , X. Liu , W.‐L. Bai , Y.‐S. Liu , Z.‐P. Cai , Q. Zhang , C. Zhao , K.‐X. Wang , J.‐S. Chen , Energy Storage Mater. 2020, 25, 52.

[267] S. H. Gillani , M. Sohail , L. A. El Maati , R. Altuijri , R. Zairov , M. F. Nazar , I. Ahmad , J. Alloys Compd. 2024, 976, 172931.

[268] S. N. Flesher , J. E. Downey , J. M. Weiss , C. L. Hughes , A. J. Herrera , E. C. Tyler‐Kabara , M. L. Boninger , J. L. Collinger , R. A. Gaunt , Science 2021, 372, 831. 34016775 10.1126/science.abd0380 PMC8715714

[269] X. Gu , Z. Cao , A. Jolfaei , P. Xu , D. Wu , T.‐P. Jung , C.‐T. Lin , IEEE/ACM Trans. Comput. Biol. Bioinform. 2021, 18, 1645. 33465029 10.1109/TCBB.2021.3052811

[270] Z. Wang , J. Pan , R. Yuan , M. Chen , X. Guo , S. Zhou , Nano Lett. 2023, 23, 6544. 37401457 10.1021/acs.nanolett.3c01567

[271] D. A. Godoy , A. A. Rabinstein , Curr. Opin. Crit. Care 2022, 28, 111. 35034077 10.1097/MCC.0000000000000914

[272] A. Ramos‐Murguialday , D. Broetz , M. Rea , L. Läer , Ö. Yilmaz , F. L. Brasil , G. Liberati , M. R. Curado , E. Garcia‐Cossio , A. Vyziotis , Ann. Neurol. 2013, 74, 100. 23494615 10.1002/ana.23879 PMC3700597

[273] C. E. Bouton , A. Shaikhouni , N. V. Annetta , M. A. Bockbrader , D. A. Friedenberg , D. M. Nielson , G. Sharma , P. B. Sederberg , B. C. Glenn , W. J. Mysiw , Nature 2016, 533, 247. 27074513 10.1038/nature17435

[274] Y. Wang , X. Gao , B. Hong , C. Jia , S. Gao , IEEE Eng. Med. Biol. Mag. 2008, 27, 64. 10.1109/MEMB.2008.923958 18799392

[275] R. Singh , N. K. Rai , A. Gupta , S. Chouhan , A. Joshi , M. Goyal , Int. J. Neurosci. 2023, 1, 2269472. 10.1080/00207454.2023.2269472 37812033

[276] E. W. Sellers , D. J. Krusienski , D. J. McFarland , T. M. Vaughan , J. R. Wolpaw , Biol. Psychol. 2006, 73, 242. 16860920 10.1016/j.biopsycho.2006.04.007

[277] K. Suefusa , T. Tanaka , IEEE Trans. Biomed. Eng. 2017, 65, 2119. 29989946 10.1109/TBME.2017.2785412

[278] S. Nagel , M. Spüler , Sci. Rep. 2019, 9, 8269. 31164679 10.1038/s41598-019-44645-x PMC6547849

[279] F. Iwane , A. Sobolewski , R. Chavarriaga , J. D. R. Millán , iScience 2023, 26, 107524. 37636067 10.1016/j.isci.2023.107524 PMC10448161

[280] L. E. Osborn , A. Dragomir , J. L. Betthauser , C. L. Hunt , H. H. Nguyen , R. R. Kaliki , N. V. Thakor , Sci. Rob. 2018, 3, eaat3818. 10.1126/scirobotics.aat3818 PMC7051004 32123782

[281] K. Zhen , S. Zhang , X. Tao , G. Li , Y. Lv , L. Yu , npj Park. Dis. 2022, 8, 146. 10.1038/s41531-022-00418-4 PMC9622812 36316416

[282] X. Zhang , Z. Ma , H. Zheng , T. Li , K. Chen , X. Wang , C. Liu , L. Xu , X. Wu , D. Lin , Ann. Transl. Med. 2020, 8, 712. 32617332 10.21037/atm.2019.11.109 PMC7327323

[283] C. Hughes , A. Herrera , R. Gaunt , J. Collinger , Handb. Clin. Neurol. 2020, 168, 163. 32164851 10.1016/B978-0-444-63934-9.00013-5

[284] L. Berthouze , A. Tijsseling , J. Three Dimens. Images 2002, 16, 141.

[285] C. F. Anderson , R. W. Chakroun , M. E. Grimmett , C. J. Domalewski , F. Wang , H. Cui , Nano Lett. 2022, 22, 4182. 35522052 10.1021/acs.nanolett.2c00967 PMC9844543

[286] P. R. Roelfsema , D. Denys , P. C. Klink , Trends Cogn. Sci. 2018, 22, 598. 29729902 10.1016/j.tics.2018.04.001

[287] L. Brown , J. van de Molengraft , R. F. Yazicioglu , T. Torfs , J. Penders , C. Van Hoof , in *Proc. Annu. Int. Conf. IEEE Eng. Med. Biol. Soc*., IEEE, Buenos Aires, Argentina 2010, 4197. 10.1109/IEMBS.2010.5627393 21096892

[288] Quasar , http://www.quasarusa.com/products_dsi.htm (accessed June 2022).

[289] Y. Shi , F. Wang , J. Tian , S. Li , E. Fu , J. Nie , R. Lei , Y. Ding , X. Chen , Z. L. Wang , Sci. Adv. 2021, 7, eabe2943. 33536215 10.1126/sciadv.abe2943 PMC7857682

[290] X. Yu , Z. Xie , Y. Yu , J. Lee , A. Vazquez‐Guardado , H. Luan , J. Ruban , X. Ning , A. Akhtar , D. Li , Nature 2019, 575, 473. 31748722 10.1038/s41586-019-1687-0

[291] R. Kumar , U. Mehta , P. Chand , Procedia Comput. Sci. 2017, 105, 264.

