# Supplementary material for: Neural interfaces: Bridging the brain to the world beyond healthcare
Source: Exploration (Beijing). 2024 Mar 14;4(5):20230146. doi: 10.1002/EXP.20230146 (PMC11491314; doi:10.1002/EXP.20230146)
Supplement: Supplementary file 1 — Supporting Information [file EXP2-4-20230146-s001.docx]

Supporting Information

**Neural interfaces: Bridging the brain to the world beyond healthcare**

*Shumao Xu,* Yang Liu, Hyunjin Lee, Weidong Li**

| **Table S1.** **Summary of different algorithms in neural signal decoding for potential smart city applications** | | | | | |
| --- | --- | --- | --- | --- | --- |
| **Method** | **Input** | **Output** | **Advantages** | **Limitations** | **Applications** |
| Filter Band Common Spatial Pattern (FBCSP) | Raw EEG/ECoG/MEA | Feature vector | Captures multiple frequency bands, more accurate than CSP | Risk of overfitting, specific to individual | Neuro-rehabilitation, gaming, and virtual reality |
| Sparse FBCSP | Raw EEG/ECoG/MEA | Sparse feature vector | Simplifies model and interpretable | Sparsity constraint complexities, intensive | Neuro-rehabilitation, adaptive interfaces |
| Independent Component Analysis (ICA) | Voltage amplitude (EEG/ECoG/MEA) | Pure signals | Artifact reduction, improves signal-to-noise ratio | Black-box nature | Neurology, cognitive neuroscience |
| Linear Discriminant Analysis (LDA) | Processed EEG/ECoG/MEA | Classification labels | Simplicity, and effectiveness for linearly separable data | Assumes Gaussian distribution, outlier sensitivity | Limb/robot control, exoskeletons, neuro-prosthetics, communication aid |
| Support Vector Machine (SVM) | Preprocessed EEG/ECoG/DBS/MEA | Classification labels | Robust to noise, high-dimensional data handling | Kernel choice and overfitting risk | Prosthetic control, rehabilitation |
| Long Short-Term Memory (LSTM) | Time-series EEG/ECoG/DBS/MEA | Sequence, labels | Captures long-term sequence dependencies | Risk of overfitting on small datasets | Time-series prediction, natural language processing |
| Recurrent Neural Networks (RNN) | Time-series EEG/ECoG/DBS/MEA | Sequence, labels | Exceptional at modeling sequence data | Gradient issues, inferior to LSTM for long-term dependencies | Real-time control and temporal pattern recognition |
| Convolution Neural Networks (CNN) | Raw/processed EEG/ECoG/MEA | Feature maps, labels | Spatial pattern recognition | Large dataset requirement, sensitive to hyperparameter choices | Image tasks, spatial pattern classification |
| Deep Neural Networks (DNN) | Raw/processed EEG/ECoG/DBS/MEA | Various labels, continuous values | Versatile, complex data handling, self-learning | Overfitting risk, complex, time-consuming | Neuro-prosthetics, communication, and rehabilitation |
| Deep Belief Networks (DBN) | Raw/processed EEG/ECoG/MEA | Various class labels, continuous values | Pre-training benefits, complex representation learning, and reduced datasets need | Overfitting risk | Neuro-prosthetics, rehabilitation |
